# Oncogenic ETS fusions promote DNA damage and proinflammatory responses via pericentromeric RNAs in extracellular vesicles

**DOI:** 10.1172/JCI169470

**Published:** 2024-03-26

**Authors:** Peter Ruzanov, Valentina Evdokimova, Manideep C. Pachva, Alon Minkovich, Zhenbo Zhang, Sofya Langman, Hendrik Gassmann, Uwe Thiel, Marija Orlic-Milacic, Syed H. Zaidi, Vanya Peltekova, Lawrence E. Heisler, Manju Sharma, Michael E. Cox, Trevor D. McKee, Mark Zaidi, Eve Lapouble, John D. McPherson, Olivier Delattre, Laszlo Radvanyi, Stefan E.G. Burdach, Lincoln D. Stein, Poul H. Sorensen

**Affiliations:** 1 Ontario Institute for Cancer Research, Toronto, Ontario, Canada.; 2 Department of Molecular Oncology, British Columbia Cancer Research Centre and; 3 Department of Pathology and Laboratory Medicine, University of British Columbia, Vancouver, British Columbia, Canada.; 4 Department of Pediatrics, Children’s Cancer Research Center, Kinderklinik München Schwabing, TUM School of Medicine and Health, Technical University of Munich, Munich, Germany.; 5 Vancouver Prostate Centre, Vancouver, British Columbia, Canada.; 6 STTARR Innovation Centre, Radiation Medicine Program, Princess Margaret Cancer Centre, University Health Network, Toronto, Ontario, Canada.; 7 Pathomics Inc., Toronto, Ontario, Canada.; 8 Department of Medical Biophysics, University of Toronto, Toronto, Ontario, Canada.; 9 Unité Génétique Somatique (UGS), Institut Curie, Centre Hospitalier Paris, France.; 10 Department of Biochemistry and Molecular Medicine, University of California Davis Comprehensive Cancer Center, Sacramento, California, USA.; 11 Diversity and Plasticity of Childhood tumors, INSERM U830, Institut Curie Research Center, PSL Research University, Paris, France.; 12 Department of Immunology, University of Toronto, Toronto, Ontario, Canada.; 13 CCC München Comprehensive Cancer Center, DKTK German Cancer Consortium, Munich, Germany.; 14 Institute of Pathology, Translation Pediatric Cancer Research Action, School of Medicine, Technical University of Munich, Munich, Germany.; 15 Department of Molecular Genetics, University of Toronto, Toronto, Ontario, Canada.

**Keywords:** Inflammation, Innate immunity

## Abstract

Aberrant expression of the E26 transformation-specific (ETS) transcription factors characterizes numerous human malignancies. Many of these proteins, including EWS:FLI1 and EWS:ERG fusions in Ewing sarcoma (EwS) and TMPRSS2:ERG in prostate cancer (PCa), drive oncogenic programs via binding to GGAA repeats. We report here that both EWS:FLI1 and ERG bind and transcriptionally activate GGAA-rich pericentromeric heterochromatin. The respective pathogen-like *HSAT2* and *HSAT3* RNAs, together with *LINE*, *SINE*, *ERV,* and other repeat transcripts, are expressed in EwS and PCa tumors, secreted in extracellular vesicles (EVs), and are highly elevated in plasma of patients with EwS with metastatic disease. High human satellite 2 and 3 (*HSAT2,3*) levels in EWS:FLI1- or ERG-expressing cells and tumors were associated with induction of G2/M checkpoint, mitotic spindle, and DNA damage programs. These programs were also activated in EwS EV-treated fibroblasts, coincident with accumulation of *HSAT2,3* RNAs, proinflammatory responses, mitotic defects, and senescence. Mechanistically, *HSAT2,3*-enriched cancer EVs induced cGAS-TBK1 innate immune signaling and formation of cytosolic granules positive for double-strand RNAs, RNA-DNA, and cGAS. Hence, aberrantly expressed ETS proteins derepress pericentromeric heterochromatin, yielding pathogenic RNAs that transmit genotoxic stress and inflammation to local and distant sites. Monitoring HSAT2,3 plasma levels and preventing their dissemination may thus improve therapeutic strategies and blood-based diagnostics.

## Abstract 2

Transcriptional activation of pericentromeric heterochromatin driven by EWS::FLI1 in Ewing sarcoma and TMPRSS2 fusion proteins in prostate cancer yield pathogenic RNAs, which transmit genotoxic stress.

## Graphical abstract

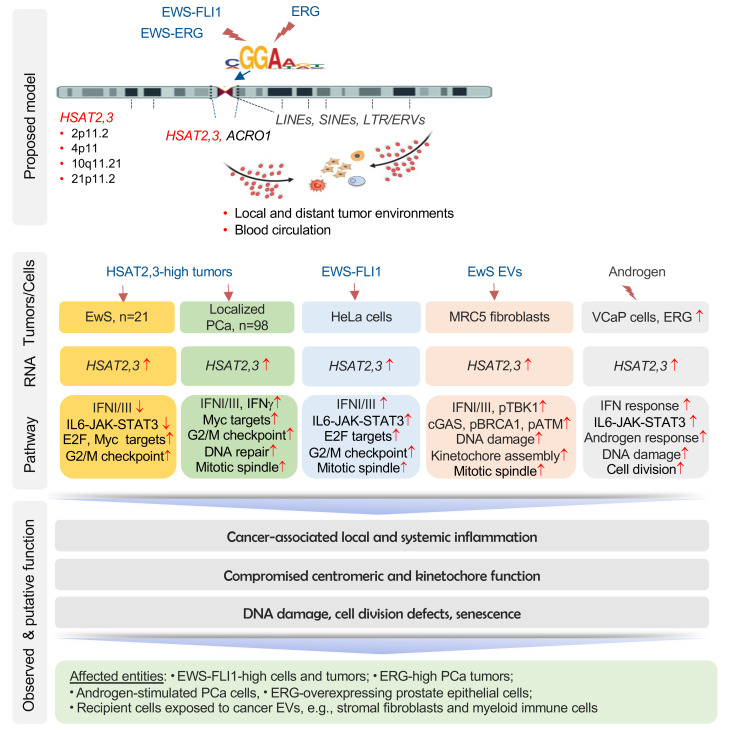

## Introduction

Ewing sarcoma (EwS) is a poorly differentiated and highly aggressive childhood and adolescent malignancy of bone and soft tissues, thought to originate from primitive bone marrow-derived mesenchymal stem cells (1, 2). Unlike the majority of adult cancers, EwS has extremely low mutation rates and is characterized by single chromosomal translocations, most frequently t(11;22) (q24;q12) and t(21;22) (q22;q12), which are found in approximately 85% and 10% of EwS cases, respectively (2–4). The resultant fusion proteins, EWS:FLI1 and EWS:ERG, consist of the N-terminal transactivation domain of the RNA-binding protein, called EWS, linked to the C-terminal DNA-binding domains of FLI1 or ERG (1–3). Both FLI1 and ERG belong to the E26 transformation-specific (ETS) transcription factor family and exhibit high degree of similarity, including virtually identical DNA-binding domains (5, 6). Aside from EwS, ETS factors are also fused or aberrantly expressed in many adult solid and hematological malignancies, including melanoma, leukemia, and carcinomas of prostate, breast, ovaries, lungs, pancreas, and colon (5). In prostate cancer (PCa), up to 50% of cases are characterized by *TMPRSS2:ERG* gene fusions, in which ERG expression is driven by the androgen-responsive *TMPRSS2* promoter (5, 7, 8).

Both EWS:FLI1 and EWS:ERG in EwS, and aberrantly expressed ERG in PCa can bind to GGAA repeats in the promoters and enhancers of coding genes and within microsatellites, causing widespread chromatin remodeling and transcriptional rewiring (5, 6, 9–13). Despite their histogenetic differences, EwS and PCa display many similarities, including evidence of genotoxic stress, such as impaired DNA damage repair pathways, accumulation of R-loops and enhanced sensitivity to poly (ADP-ribose) polymerase (PARP) inhibitors, similar to BRCA1/2-deficient breast cancer (5, 14–16). Lastly, both EwS and PCa belong to the class of immunologically cold solid tumors and are characterized by persistent inflammation and the immunosuppressive tumor microenvironment (TME), which facilitates tumor progression and resistance to chemo and immunotherapy (17). Identifying and targeting mechanisms driving genotoxic stress and inflammation in these cancers may improve clinical outcomes.

Our prior work showed that extracellular vesicles (EVs) released by EwS cells inhibit T cell activation and induce proinflammatory phenotypes in CD14^+^ and CD33^+^ blood monocytes (18, 19). Here, we demonstrate that this effect is likely mediated by repeat RNAs, including human satellite 2 and 3 (*HSAT2,3*), long and short interspersed elements (*LINEs* and *SINEs*), and endogenous retroviruses (*LTR/ERVs*). We show that *HSAT2,3* expression is directly driven by EWS:FLI1 and ERG in EwS and PCa cells, respectively, and may represent one of the earliest events triggering DNA damage and inflammation in tumor cells and the TME.

## Results

### EwS EVs are enriched with retroelement and pericentromeric RNAs.

Our previous finding that EwS EVs induce antiviral innate immune pathways in healthy donor-derived monocytes (18) prompted us to investigate the potential role of the RNAs they carry. Total EV preparations were purified from the conditioned medium of a panel of EwS cell lines by filtration and differential centrifugation (Supplemental Figure 1A; supplemental material available online with this article; https://doi.org/10.1172/JCI169470DS1). Their purity was confirmed by nanoparticle tracking, RNA, and immunoblotting, and was consistent with the characteristics of small EVs (20–22), including their size (40–200 nm), enrichment with small (under 200 nt) RNAs, and lack of major cellular ribosomal 18S and 28S RNAs (Supplemental Figure 1, B and C). Compared with parental cells, purified EVs were also enriched with classical exosome markers Syntenin 1 and CD81, weakly positive for microvesicle markers Annexin A1 and ARRDC1, and negative for common cellular contaminants Calnexin, Hsp90, Histone H2A, β-Tubulin and the autophagosome marker LC3B (Supplemental Figure 1D).

**Figure 1:**
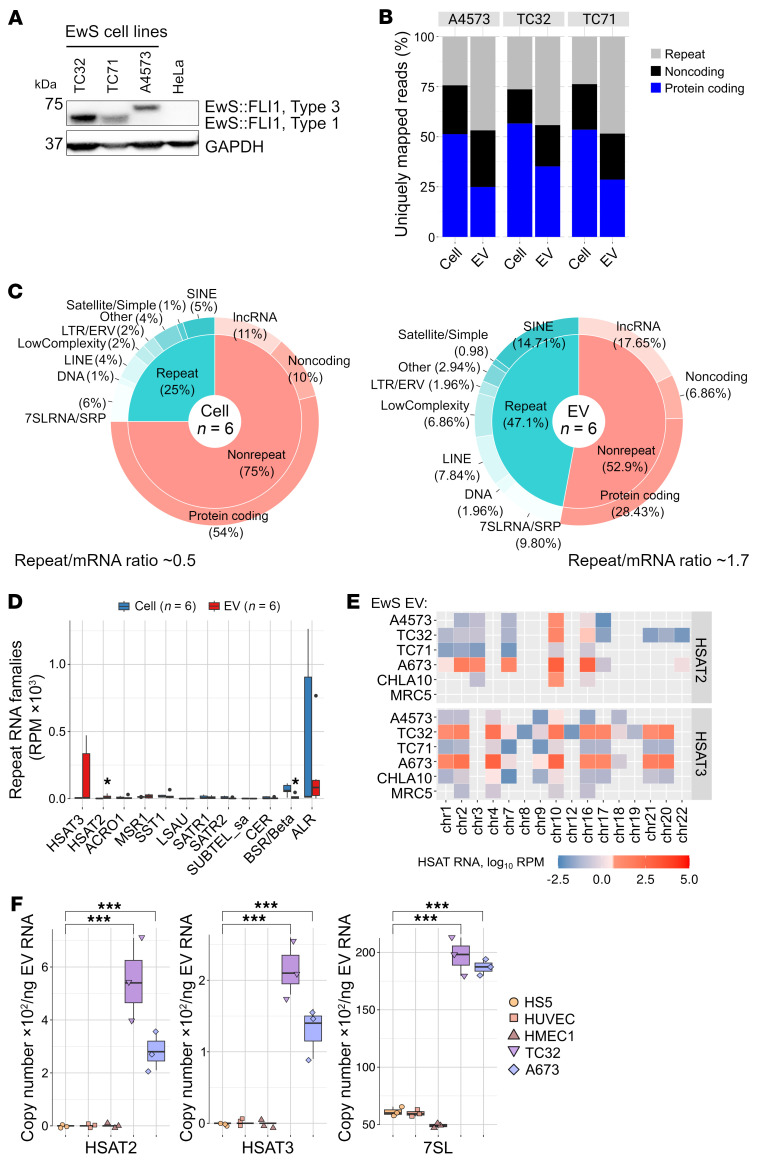
EwS EVs are enriched with retroelement and pericentromeric RNAs. (**A**) Immunoblotting detection of the endogenous EWS:FLI1 protein in EwS and HeLa cell extracts using FLI1 antibodies and GAPDH as a loading control. (**B**) Percentage of RNA-Seq reads mapped to “Repeats” (RepeatMasker-annotated elements), “Noncoding” (pseudogenes, long and small ncRNAs), and “Protein-coding” (mRNAs, 5′ and 3′ UTRs, and introns) in each EwS cell line and the respective EVs from 2 biological replicates. (**C**) Pie charts representing averaged values for each repeat and nonrepeat category detected in EwS cells and EVs from **B**. Comparisons made between repeat RNA content in EVs versus cells; *P* = 0.032, Wilcoxon signed-rank test. (**D**) Representation of each satellite family member, reads per million mapped reads (RPM). Comparisons EVs versus cells; *P < 0.01, Wilcoxon test. (**E**) Chromosomal localization of HSAT2,3 RNA-Seq reads detected in EVs from EwS and MRC5 cell lines. Expression values were log_10_-scaled. Each square represents an average expression from 2 replicates. Gray color indicates no reads. (**F**) RT-ddPCR of indicated RNAs in EVs from nontransformed (HS-5, HUVEC, HMEC-1) and EwS (TC32 and A673) cell lines. Data are mean ± SEM; comparisons to HS-5 EVs, ***P < 0.001, 2-way ANOVA.

We next analyzed the EV RNA contents by whole transcriptome RNA -Seq, initially testing 3 EwS cell lines positive for type 1 (TC32, TC71) or type 3 (A4573) EWS:FLI1 fusion proteins (Figure 1A and Supplemental Table 1). RNA-Seq analysis of EV RNAs compared with the respective parental cells showed an approximately 2-fold decrease of protein coding transcripts and a robust enrichment with *7SL/SRP*, *LINE*, *SINE,* and *LTR/ERV* and satellite repeat RNAs, with repeat RNA/mRNA ratios of 1.7 and 0.5 in EVs and cells, respectively (Figure 1, B and C). These patterns were characteristic of EVs from all 3 EwS cell lines (Figure 1B), and were previously reported in other tumor EVs, including melanoma, brain, and breast cancers (23–25).

Notably, *HSAT2,3* RNAs were almost exclusively detected in EVs but not in the corresponding parental cell lines, which instead exhibited high levels of *ALR* RNA from large centromeric α satellite repeats (Figure 1D). These RNAs originated from the GGAAT-rich pericentromeric regions defined by tandemly repeated approximately 23- to 26-bp consensus motifs (*HSAT2*) or by highly divergent GGAAT/ATTCC simple repeats (*HSAT3*) (26–28). Despite their sequence variability, we were able to uniquely map approximately 60% of *HSAT2,3* reads using Burrows-Wheeler Aligner (BWA) default parameters, with *HSAT2* predominantly mapping to 10q11.21 and 16q11.2, and *HSAT3* to multiple pericentromeric regions (Figure 1E). Using additional cell lines, we found that EVs from A673 and TC32 EwS cells were highly enriched with *HSAT2,3* RNAs, while EVs from normal diploid MRC5 fibroblasts were negative (Figure 1E). This was also verified by reverse transcription droplet-based digital polymerase chain reaction (RT-ddPCR), which showed high abundance of *HSAT2,3* and *7SL/SRP* RNAs in EVs from TC32 and A673 cells, but not from nontransformed HS-5 fibroblast-like stromal cells or HMEC-1 and HUVEC endothelial cells (Figure 1F). Together, these data uncover extensive expression of locus-specific *HSAT2,3* RNAs in EwS cells, and their enrichment in EVs, conjointly with a variety of retroelement and other repeat RNAs.

### Repeat RNAs are the most abundant species in plasma of patients with EwS.

**Figure 2:**
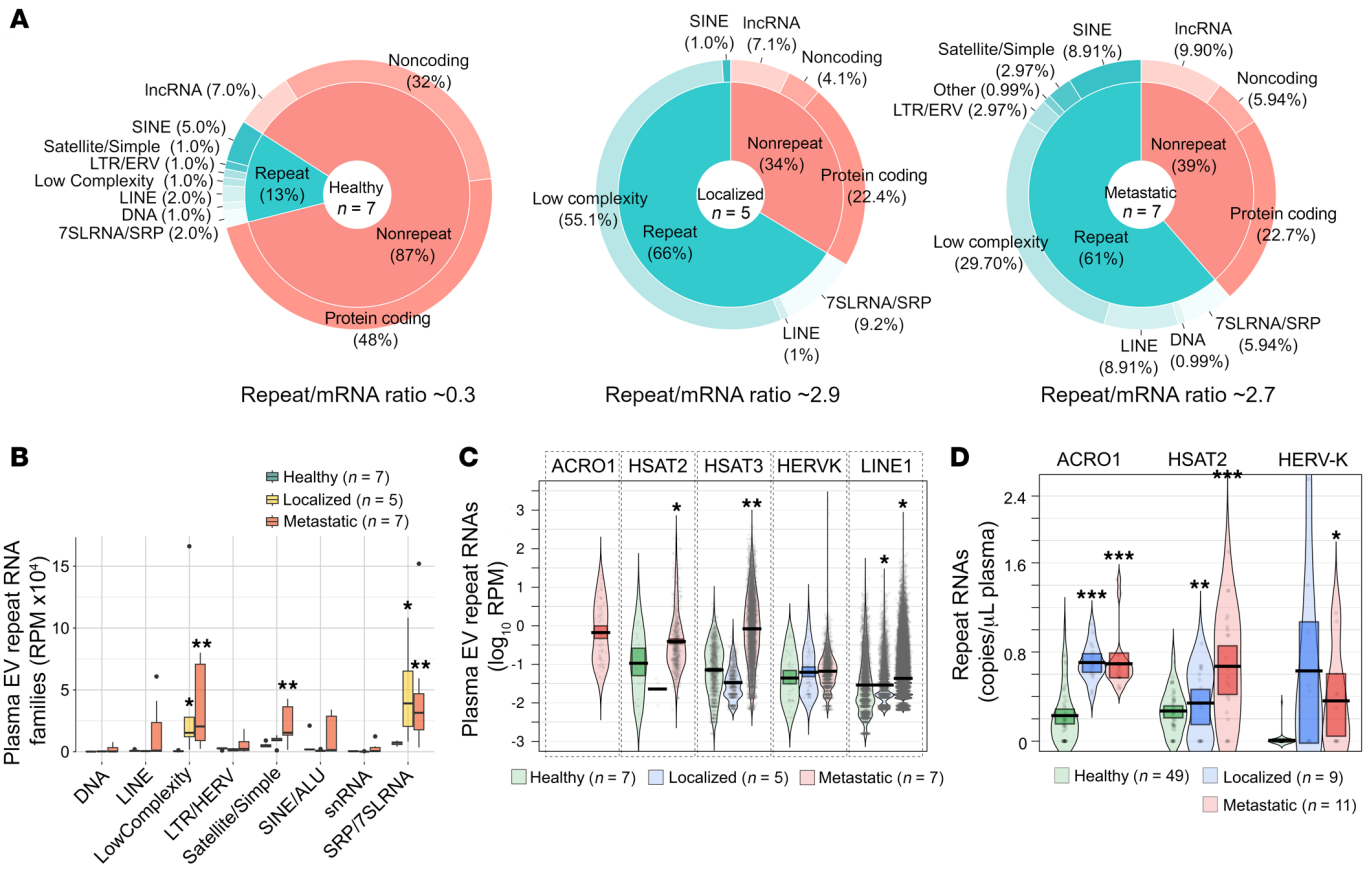
Repeat RNAs are the most abundant species in blood plasma of patients with EwS. (**A**) RNA-Seq of plasma EV RNAs from patients with EwS (EW cohort; *n* = 12) and age-matched healthy donors (*n* = 7). Pie charts represent averaged values for each repeat and nonrepeat category. Comparisons made between patients with EwS versus healthy donors; *P* = 0.012, Wilcoxon signed-rank test. (**B**) RepeatMasker-annotated families from **A** shown as mean RPM ± SEM; patients with EwS versus healthy donors, *P < 0.05, **P < 0.01, Wilcoxon signed-rank test. (**C**) Violin plots of representative RNAs from **B**; patients with EwS versus healthy donors, *P < 1.36×10^–11^, **P < 2.00×10^–41^. Note that *ACRO1* was not detected in healthy donors and patients with localized disease. (**D**) RT-ddPCR of indicated RNAs in plasma of patients with EwS (TUM cohort, *n* = 20) versus healthy donors (*n* = 49); *P < 0.05, **P = 0.0002, ***P < 2.00×10^–8^. Variance between groups was analyzed with the Welch F test, *P* = 4.27×10^–12^ (*ACRO1*), *P* = 0.0038 (*HSAT2*) and *P* = 0.0158 (*HERV-Kenv*). *P* values in **C** and **D** were adjusted for multiple comparisons using BH method.

To establish the potential clinical relevance of the above findings, we purified and characterized EVs from plasma of newly diagnosed therapy-naive patients with EwS with localized or metastatic disease (designated the EW cohort) and age-matched healthy donors (Supplemental Table 2). Purified plasma EVs were within a standard size range (40–200 nm), positive for CD63 and CD81 exosome markers, and enriched with small (under 200 nt) RNAs (Supplemental Figure 2, A–D). Whole transcriptome RNA-Seq analysis of uniquely mapped nonribosomal reads revealed significant differences between patients with EwS (*n* = 12) and healthy age-matched donors (*n* = 7); of total RNA content, approximately 61%–66% of RNAs in plasma EVs from patients with EwS were derived from repeats, compared with approximately 13% in healthy donors (*P* = 0.012, Wilcoxon signed-rank sum test; Figure 2A). Similar to EVs purified from EwS cell lines, 7SL/SRP, simple, and low complexity repeat RNAs were the most abundant repeat RNAs in plasma EVs of all patients with EwS (Figure 2B). Retroelement (*LINE*, *SINE*, and *LTR/ERV*) and pericentromeric (*ACRO1* and *HSAT2,3*) RNAs were highly upregulated, especially in patients with metastasis (Figure 2, B and C). These results were validated by RT-ddPCR using the additional plasma EV specimens from 20 patients with EwS (designated the TUM cohort) and 49 age-matched healthy donors (Supplemental Table 2), which showed a significant increase of *ACRO1*, *HSAT2*, and *HERV-K* envelope (HERV-Kenv) RNAs in plasma of patients with EwS versus healthy donors (*P* < 0.05, Wilcoxon signed-rank test; Figure 2D). Overall, EVs from patients with EwS exhibited higher repeat RNA/mRNA ratio (approximately 2.7–2.9 versus 0.3 in healthy donors), revealing a striking difference between patients with EwS and healthy donors.

### HSAT2,3 are expressed in EwS tumors.

**Figure 3:**
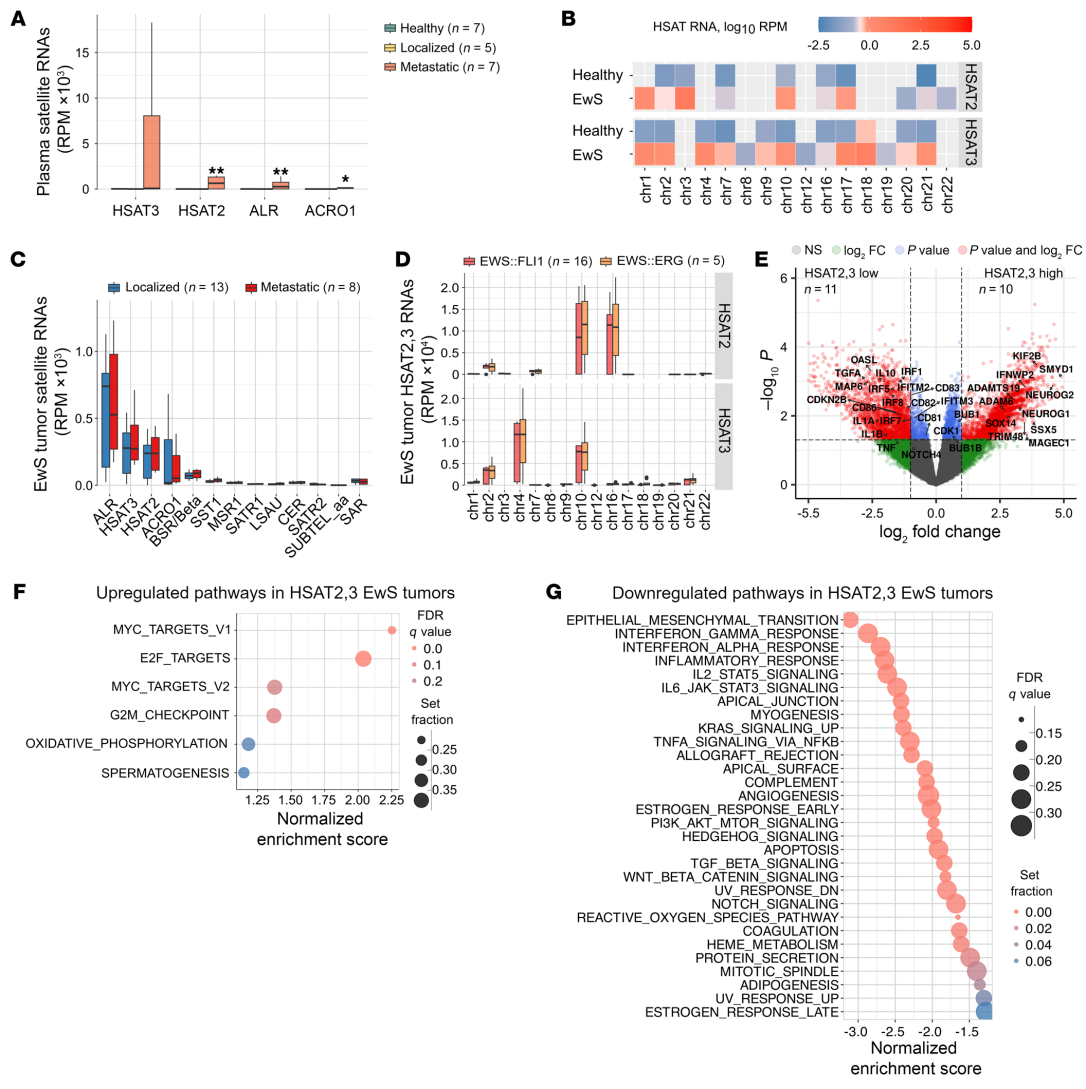
Elevated *HSAT2,3* expression in EwS tumors is associated with upregulation cell cycle checkpoint and downregulation of immune response genes. (**A**) Representation of satellite RNAs in plasma of patients with EwS versus healthy donors. Data are mean RPM ± SEM; *P < 0.05, **P < 0.01, Wilcoxon signed-rank test. (**B**) Chromosomal distribution of uniquely mapped *HSAT2,3* reads in plasma of patients with localized or metastatic disease. Cumulative read counts are shown. (**C**–**G**) Analysis of EwS tumor data sets (*n* = 21). Representation of satellite RNAs (**C**) and chromosomal distribution of uniquely mapped *HSAT2,3* RNA-Seq reads in EwS tumors positive for EWS:FLI1 or EWS:ERG (**D**). Data in **C** and **D** are mean RPM ± SEM. (**E**) Volcano plot, log_2_ FC versus –log_10_ *P* value of differentially expressed genes in *HSAT2,3-*high versus -low EwS tumors. Red dots represent genes which passed *P* ≤ 0.05 thresholds and changed >2-fold. (**F** and **G**) GSEA of up or downregulated pathways in *HSAT2,3*-high EwS tumors, ranked based on log_2_ FC from **E**. Circle size indicates gene set size, and circle color indicates the FDR-adjusted *q* value.

Among different satellite RNAs, *HSAT2* and, especially, *HSAT3* were the most abundant species in plasma EVs from patients with EwS with metastatic disease (Figure 3A). Similar to EwS EVs from cell lines, *HSAT2,3* reads from plasma EVs of patients with EwS were mapped to multiple genomic loci including 1q12, 2p11.2, 4p11, 10q11.21, and 21p11.2, and covered both sense and antisense strands of *HSAT2,3* arrays, adjacent to the centromeric α satellite repeat ALR (Figure 3B and Supplemental Figure 3). Analysis of published RNA-Seq tumor data sets from 21 patients with EwS (29) confirmed the expression of *HSAT2,3* RNAs in primary and metastatic tumors, including cases with EWS:FLI1 (*n* = 16) and EWS:ERG (*n* = 5) fusions, and their origins from the 4p11, 10q11.21, and 16q11.2 HSAT2,3 loci (Figure 3, C and D). Differential gene expression analysis revealed a sharp distinction between *HSAT2,3*-high versus -low tumors, with 2,131 mRNAs upregulated and 570 downregulated (log_2_ fold change ≥ 2, FDR < 0.25; Figure 3E). Gene set enrichment analysis (GSEA) of *HSAT2,3*-high versus -low tumors showed a significant enrichment with cell cycle and proliferation pathways (hallmark *MYC*, *E2F*, G2/M checkpoint; FDR < 0.2; Figure 3F) and downregulation of inflammatory and IFN pathways (FDR < 0.05; Figure 3G).

Using commercial probes, we next analyzed *HSAT2* and *HERV-K* RNA expression in matching primary and metastatic tumors from 3 patients with EwS by ViewRNA in situ hybridization (ISH). In all 3 cases, *HSAT2* was significantly upregulated in metastatic sites compared with primary tumors (*P* < 0.05, unpaired 2-tailed equal variance *t* test), while *HERV-K* showed an opposite trend (Supplemental Figure 4, A and B). Overall, *HERV-K* levels were substantially higher than those of *HSAT2* RNAs, which could be due to the preferential secretion of *HSAT2* in EVs or due to detection limitations, given that only a fraction of these highly polymorphic RNAs could be detected with any particular probe. Hence, *HSAT2,3* RNAs are expressed in primary and metastatic EwS tumors with EWS:FLI1 and EWS:ERG fusions, and their expression is linked to cell cycle defects and reduced immune responsiveness.

### EWS:FLI1 preferentially binds to HSAT3 pericentromeric chromatin and activates HSAT2,3 RNA expression in non-EwS cells.

**Figure 4:**
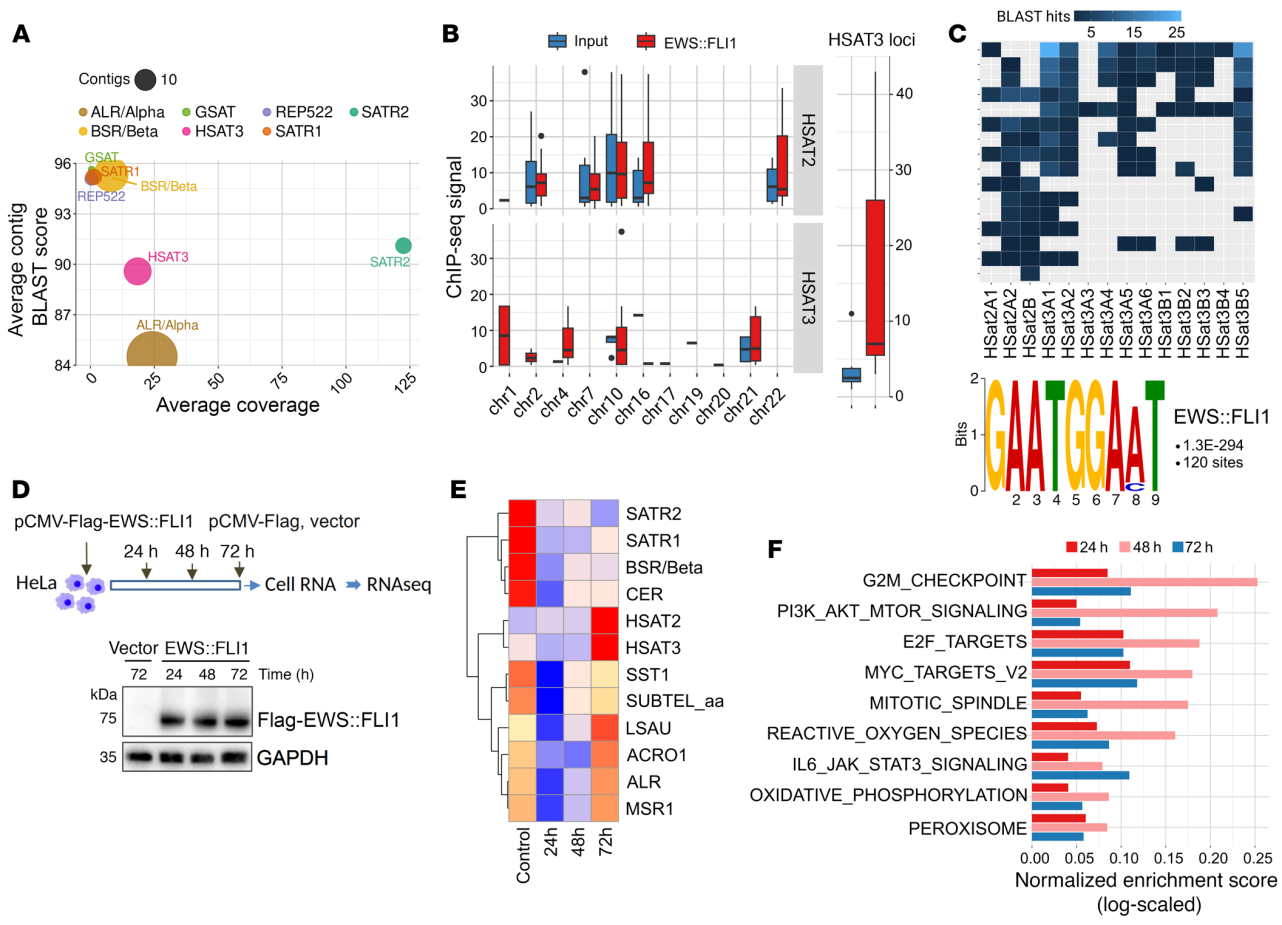
EWS:FLI1 preferentially binds to *HSAT3* pericentromeric chromatin and activates *HSAT2,3* expression in non-EwS cells. (**A**) Analysis of EWS:FLI1 binding sites in centromeric regions by alignment with 200–700 nt contigs assembled from the unmapped EWS:FLI1 ChIPseq reads; number of binding peaks overlapping a particular repeat element is reflected by bubble size. Peaks of highest quality (average MACS2 peak score > 5) are shown. (**B**) Chromosomal distribution of the *HSAT* contigs (left panel) and total number of the identified *HSAT3* loci (right panel) from the EWS:FLI1 ChIP-Seq data set versus input fractions (*P* = 0.0037, Wilcoxon rank-sum test). Contigs with alignment identity ≥ 95% were analyzed. (**C**) Identification of EWS:FLI1 binding motifs in pericentromeric regions by alignment to the 24-mer *HSAT2,3* motif libraries (top) and highest-scoring recurring sequence motif (bottom). (**D**) Outline of HeLa transient transfection experiments (top) and immunoblotting using Flag antibodies and GAPDH as a loading control (bottom). (**E** and **F**) Heatmap of satellite RNA expression (**E**) and pathway enrichment analysis (**F**) in the respective HeLa cells from **D**. Color in **E** indicates normalized RPM values scaled and centered by row. Each time point is average value from 2 biological replicates.

Knowing that EWS:FLI1 and EWS:ERG bind to core GGA (A/T) motifs with at least 4 consecutive GGAA repeats (11, 13), we next assessed if EWS:FLI1 can bind to GGAA-rich pericentromeric heterochromatin using published chromatin immunoprecipitation–Seq (ChIP-Seq) data sets from A673 EwS cells (10). Because pericentromeric regions are usually excluded from the analysis due to the high noise-to-signal ratio (13), we assembled the unmapped EWS:FLI1 ChIP-Seq reads into 200–700 nt contigs, to enhance selectivity and reduce false positives. This identified approximately 20 high-quality contigs mapping to *HSAT2,3* repeats on 1q12, 4p11, 10q11.21, and 21p11.2, albeit only the *HSAT3* binding sites were significantly enriched compared with the input control (*P* = 0.0037, Wilcoxon rank sum test; Figure 4, A and B). Analysis of the respective contig sequences with BLAST showed strong homology (over 95%) to previously established *HSAT2,3* subfamily-specific 24-mers (26) and identified GAATGGAAT as the highest-scoring recurring sequence motif (Figure 4C).

To further explore the possibility that EWS:FLI1 directly binds to pericentromeric heterochromatin and induces expression of *HSAT2,3* RNAs, we used HeLa cells as they are known to be *HSAT2*-negative (30, 31) and permissive for EWS:FLI1 expression (32). Transient expression of EWS:FLI1 in these cells followed by RNA-Seq showed that *HSAT2* and *HSAT3* RNAs were strongly upregulated in EWS:FLI1–expressing cells compared with vector controls, while other satellite RNAs were either downregulated (*SATR1*, *SATR2*, *BSR*, and *CER*) or not substantially changed (*SST1*, *ACRO1*, *ALR*, and *MSR1*; Figure 4, D and E). The effect was notable only at 72 hours posttransfection, despite EWS:FLI1 protein levels reaching maximum at much earlier time points (24 and 48 hours), suggesting a relatively slow transcriptional activation of *HSAT2,3* heterochromatin in these settings. Similar to *HSAT2,3*-high EwS tumors, GSEA analysis of coding transcripts in EWS:FLI1 expressing versus vector HeLa cells revealed upregulation of cell cycle and DNA damage pathways, including G2/M checkpoint, PI3K-AKT-mTOR, E2F, c-MYC, mitotic spindle, reactive oxygen species, and IL-6-JAK-STAT3 inflammatory signaling categories (Figure 4F and Supplemental Figure 5). These results identify *HSAT3* as a preferential EWS:FLI1 binding site within pericentromeric heterochromatin and demonstrate upregulation of *HSAT2,3* RNA upon transient expression of EWS:FLI1 in HeLa cells, concomitant with activation of cell cycle, DNA damage, and proinflammatory pathways.

### ERG binds to pericentromeric chromatin and activates HSAT2,3 expression and DNA damage pathways in PCa cells.

**Figure 5:**
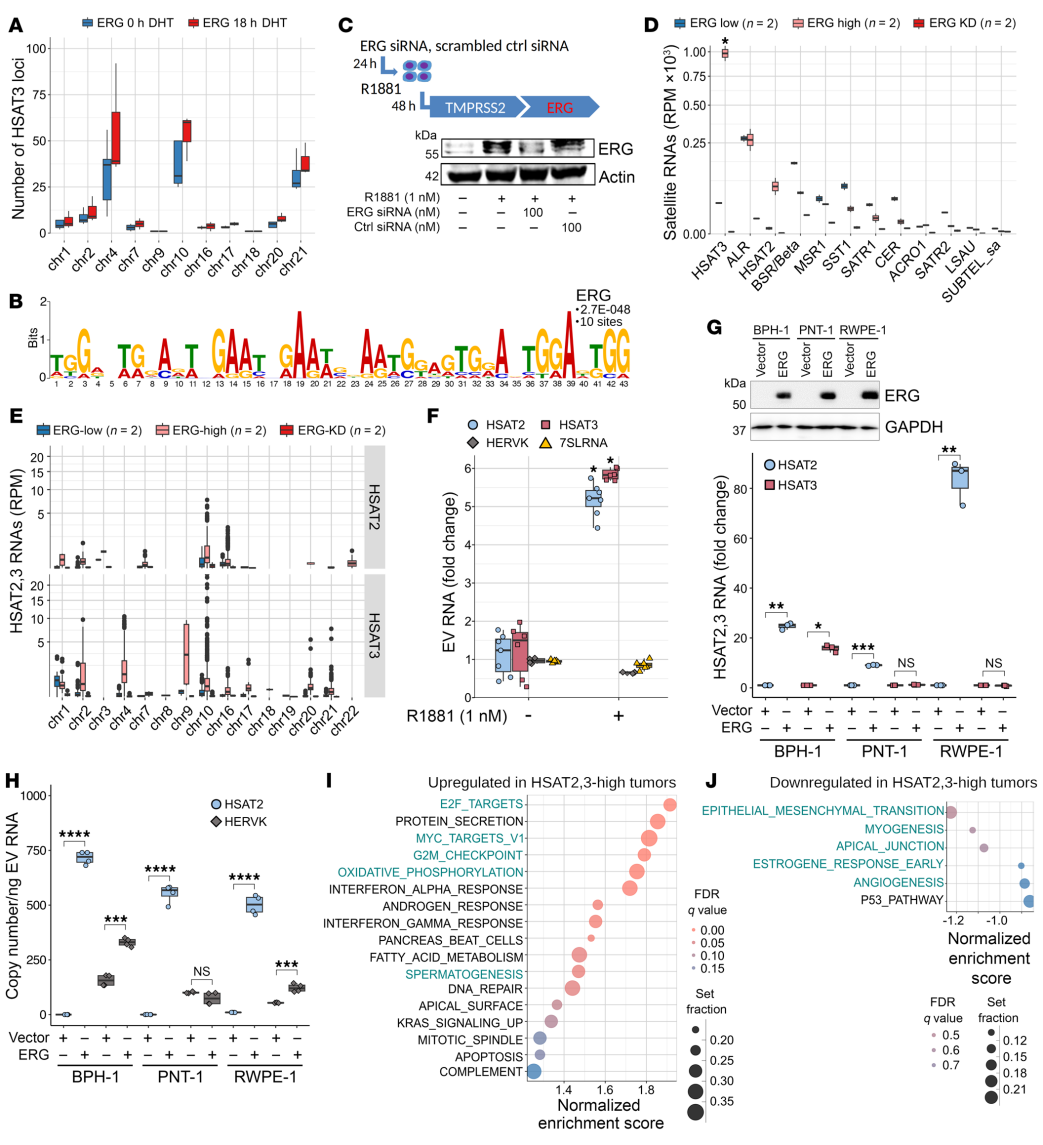
ERG binds to pericentromeric regions and activates *HSAT2,3* expression and DNA damage pathways in PCa cells. (**A**) Chromosomal distribution of the *HSAT* contigs assembled from VCaP cell-derived ERG ChIP-Seq reads. ERG-high (18 hour DHT) versus ERG-low (0 hour DHT), *P* ≤ 0.001, 2-way ANOVA. (**B**) Highest-scoring consensus motif in ERG ChIP-Seq contigs. (**C**) Experimental outline (top) and immunoblotting detection of the endogenous ERG protein in androgen-deprived (–) or R1881-stimulated VCaP cells pretreated with ERG or scrambled control (ctrl) siRNAs (bottom). (**D**) Representation of satellite RNAs in androgen-deprived (ERG-low) and R1881-stimulated (ERG-high) and ERG KD VCaP cells. (**E**) Chromosomal distribution of *HSAT2,3* RNA-Seq reads; Y-axis, square-root transformed. Each plot represents mean RPM ± SEM from 2 biological replicates. Comparisons made ERG-high versus -low cells; *P < 0.05, unpaired 2-tailed *t* test. (**F**) RT-qPCR of indicated RNAs in VCaP EVs after 48 hour treatment with R1881. Values normalized to the exogenously added spike-in *MS2* RNA ± SD (*n* = 3). Fold change relative to untreated control; *P < 0.001, unpaired 2-tailed *t* test. (**G**) Immunoblotting of benign prostate cells expressing ERG or vector control (top) and RT-qPCR of *HSAT2,3* expression in these cells (bottom). Values normalized to *GAPDH* RNA ± SEM (*n* = 2), representative of 1 of 2 independent experiments. Fold change relative to vector control; *P < 0.05, **P < 0.01, ***P < 0.001, unpaired 2-tailed *t* test. (**H**) RT-ddPCR of indicated RNAs in EVs from cells in **G**. Data are mean ± SD. Comparisons to EVs from vector control cells, ***P < 0.001, ****P < 0.0001, unpaired 2-tailed *t* test. (**I** and **J**) GSEA of hallmark pathways upregulated (**I**) or downregulated (**J**) in *HSAT2,3*-high PCa tumors (*n* = 98). Circle size indicates gene set size, and circle color indicates the FDR adjusted *q* value. Note that none of downregulated pathways reached statistical significance. Common pathways detected in PCa and EwS tumors are depicted in green.

Given the structural and functional similarities between FLI1 and ERG (5, 6) and the reported ability of ERG and other ETS factors to activate transcription in PCa cells via binding to GGAA repeats in a complex with the WT EWS protein (12), we next investigated if transcriptional activation of *HSAT2,3* pericentromeric heterochromatin may also occur in ERG-driven cancers, particularly PCa, where ERG is overexpressed in more than 50% of cases (5, 8). We used the *TMPRSS2:ERG* fusion gene-positive VCaP cell line, where endogenous ERG expression can be induced by natural (dihydrotestosterone, DHT) or synthetic (methyltrienolone, R1881) androgens via androgen receptor (AR) binding to the *TMPRSS2* promoter (7). Similar to EWS:FLI1, analysis of published ERG ChIP-Seq data sets (9) yielded *HSAT3* (but not *HSAT2*) contigs which mapped to the 4p11, 10q11.21, and 21p11.2 *HSAT2,3* loci. The occupancy of these binding sites was significantly increased in DHT-treated (18 hour DHT) ERG-high versus untreated (0hour DHT) ERG-low cells (*P* ≤ 0.001; 2-way ANOVA; Figure 5A). The top-scoring ERG-specific 43-mer contained multiple continuously repeated GAAT sequences (Figure 5B). Among lower-scoring motifs, we also identified GAATGGAAT, suggesting that ERG and EWS:FLI1 binding sites may at least partially overlap.

To examine if *HSAT2,3* expression in PCa cells was ERG-dependent, VCaP cells were transfected with ERG-targeting or scrambled control (ctrl) siRNAs followed by treatment with R1881 for 48 hours, to induce the endogenous ERG expression (Figure 5C, top panel). As expected, ERG protein levels were elevated in R1881-stimulated (ERG-high) compared with unstimulated (ERG-low) cells, and were diminished upon treatment with ERG-targeting siRNA (ERG KD; Figure 5C, bottom panel). Coincident with the ERG increase, RNA-Seq analysis showed significant upregulation of *HSAT2,3* (but not *ALR*) in ERG-high versus ERG-low VCaP cells (approximately 60-fold; *P* = 0.028, paired 2-tailed *t* test; Figure 5D), with the majority of reads mapping to the 2p11.2- and 4p11-*HSAT3* and 10q11.21-*HSAT2,3* loci (Figure 5E). Expression of *HSAT2,3* RNAs was likely driven by ERG and not by androgen, given that their levels were reduced in ERG-KD cells despite treatment with R1881 (Figure 5, D and E). For unclear reasons, other satellite RNAs were diminished in ERG-high and even more so in ERG-KD cells (Figure 5D), which may reflect their interdependent expression within centromeric chromatin (33). Both *HSAT2* and *HSAT3* RNAs were also detected in EVs and their levels increased by more than 4-fold in EVs from ERG-high versus ERG-low cells (*P* < 0.001, *t* test), while *HERV-K* and *7SL/SRP* remained unchanged (Figure 5F). As expected, differential gene expression analysis of ERG-high versus ERG-low VCaP cells identified upregulation of canonical androgen (*KLK2*, *KLK3/PSA*, *FKBP5*, *STEAP4*, *NKX3-1*, and *TMPRSS2*) and ERG (*PLAT*, *SERPINE1/PAI-1*, *ADAMTS1*, and *EZH2*) signature genes (Supplemental Figure 6A). Moreover, genes involved in proinflammatory responses and immunosuppression (*IL-10*, *IDO1*, *SOCS1*, *PDCD1/PD-1*, and *IL-6*), DNA damage repair (*BRCA1*, *H2AFX*, and *XRCC2*) and centrosome and kinetochore assembly networks (*KIF20A*, *KIF20B*, *KIF2C*, *PLK2*, *PLK3*, *CDC25B*, *CDC20*, *AURKA*, *AURKB*, *CENPL*, and *CENPU*) were also upregulated (Supplemental Figure 6, B and C). GSEA of ERG-high versus -low cells confirmed significant upregulation of numerous known PCa-associated and inflammatory pathways, including *TNFA*, *KRAS*, *WNTB-CATENIN*, and *IL-6-JAK-STAT3* networks (FDR < 0.1; Supplemental Figure 6D).

To test if *HSAT2,3* expression can be induced by ERG in nontransformed cells, we examined normal prostate (PNT1B and RWPE1) and benign prostatic hyperplasia (BPH1) cell lines transduced to express ERG or vector alone, as previously described (34). We found that, compared with vector alone, *HSAT2* levels were significantly upregulated in all 3 ERG-expressing cell lines (Figure 5G) and were also detected in EVs, along with *HERV-K* (Figure 5H). However, *HSAT3* RNAs were detected only in ERG-BPH-1 cells (Figure 5G) and not in EVs, suggesting that ERG alone is not sufficient to activate *HSAT3* expression in noncancerous prostate cells.

To examine *HSAT2,3* RNA expression in tumor settings and to identify coactivated gene expression programs, we interrogated RNA-Seq data sets from 98 patients with localized PCa who were treatment naive (35). This showed that *HSAT3* and, to a lesser extent, *HSAT2* were expressed in PCa tumors but their levels were substantially lower than those of *ALR* (Supplemental Figure 7A), resembling their expression patterns in EwS tumors (Figure 3C). Elevated levels of *HSAT2,3* RNAs positively correlated with tumor grade (Gleason score 6 versus 7–8) and a slightly increased risk of biochemical recurrence (Supplemental Figure 7, A and B), but none of them reached statistical significance. We also did not find a significant correlation with any of the most frequent somatic mutations detected in these tumors, including *ZFHX3*, *APC*, *CDKN1B*, *RNF43*, *TP53,* and *ATM* genes (Supplemental Figure 7C). Similar to EwS tumors (Figure 3F), GSEA analysis of *HSAT2,3*-high versus -low PCa tumors revealed evidence of cell cycle dysregulation and genotoxic stress, as manifested by significant upregulation of *E2F*, *MYC*, G2/M checkpoint, mitotic spindle, and DNA damage repair networks (FDR < 0.05; Figure 5I), while downregulated pathways did not reach statistical significance (Figure 5J). In contrast to EwS, however, the IFN response was significantly upregulated in *HSAT2,3*-high PCa tumors (FDR < 0.1; Figure 5I), and, thus, immunogenicity of *HSAT2,3*-expressing tumors may be cancer type/stage and context-dependent.

### HSAT RNAs exhibit pathogen-like features and are disseminated in the TME.

**Figure 6:**
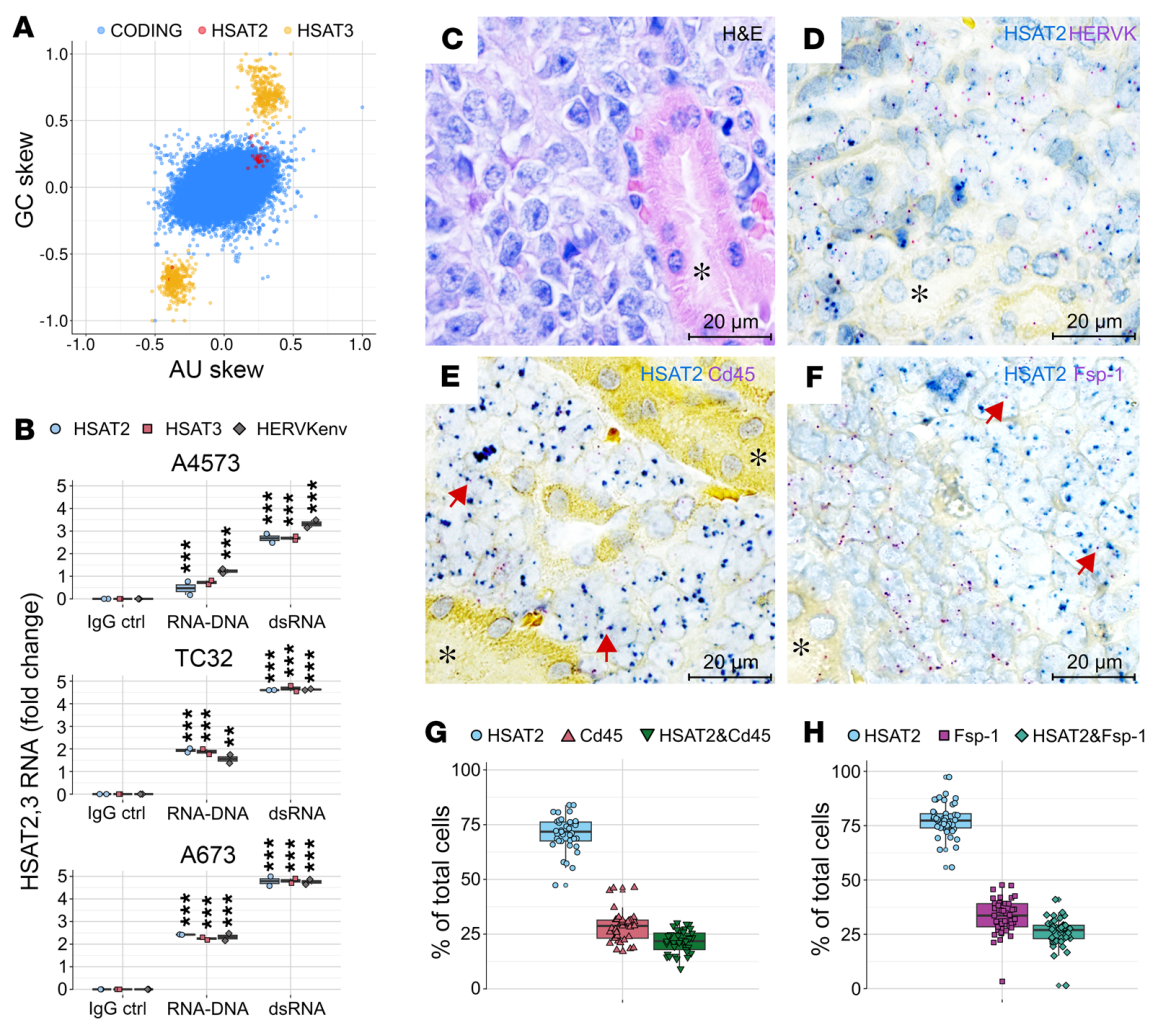
*HSAT2,3* RNAs exhibit pathogen-like features and are disseminated in the TME. (**A**) CpG or UpA motif usage in *HSAT2,3* RNAs compared with coding transcriptome. The GC and AU skew is calculated as (A-U)/(A+U) and (G-C)/(G+C) ratio, and the results are plotted in R using ggplot2 package. (**B**) RT-qPCR of RNAs immunoprecipitated from the respective CM using IgG control, S9.6 (RNA-DNA) and J2 (dsRNA) antibodies. Each box plot represents 2 technical replicates of 1 of 2 independent experiments. Values normalized to the exogenously added spike-in *MS2* RNA ± SD. FC relative to IgG control; unpaired *t* tests with BH adjustment, **P < 0.01, ***P < 0.001. (**C**) H&E staining of mouse renal subcapsular TC32 EwS tumor xenografts (**D**–**F**) ViewRNA-ISH staining with the indicated probes, counterstained with hematoxylin. Red arrows, colocalization of human HSAT2 (blue dots) with mouse Fsp-1 or Cd45 (purple dots) in a single cell. Asterisks, adjacent normal mouse kidney parenchyma. (**G** and **H**) Data quantification from **E** and **F** using Developer XD machine learning software. Data are mean ± SD. Scale bars: 20 μm.

Previous studies have indicated that *HSAT2,3* repeats in the human genome possess unusual hydrodynamic and hydrogen bonding properties (36). Additionally, *HSAT2* RNAs exhibit pathogen-like CpG and UpA motif usage and can propagate via reverse transcription and reinsertion into the genome (37, 38). We found that, indeed, *HSAT2* and, especially, *HSAT3* RNAs could be distinguished from cellular mRNAs based on asymmetrical distribution of GC and AU nucleotides (GC/AU skew; Figure 6A) and can thus be recognized by innate immune sensors as nonself. Moreover, they are likely present in EVs (but not in cells) in a double-stranded (ds) form, given equal ratios of *HSAT2,3* RNA-Seq reads in sense and antisense orientations (Supplemental Figure 8), which may further increase their immunogenicity. Indeed, using dsRNA-specific J2 antibodies, we were able to immunoprecipitate *HSAT2,3* and *HERV-K* RNAs from the conditioned medium from 3 different EwS cell lines (Figure 6B). There was a smaller but significant fraction of these RNAs immunoprecipitated along with S9.6 antibodies, suggesting the presence of RNA-DNA hybrids.

To investigate if these RNAs can be transferred to stromal cells in the TME, we used a previously developed xenograft model of EwS, where TC32 cells were implanted under the renal capsules of NOD/SCID mice (39). Xenografted TC32 tumors exhibited typical EwS morphology of small blue tumor cells with enlarged nuclei (Figure 6C). ViewRNA-ISH with commercial probes for human *HSAT2* and *HERV-K* RNAs confirmed their expression in tumor cells, some of which contained large *HSAT2*-positive nuclear aggregates (Figure 6D). Using the *HSAT2* probes in combination with probes for mouse immune cells (*Cd45*) or fibroblasts (*Fsp-1*), we identified *HSAT2*-positive mouse cells in proximity to areas of tumor beds, while mouse epithelial cells and more distant *Fsp-1^+^* fibroblasts in adjacent normal kidney tissues were negative (Figure 6, E and F). Quantitative image analysis showed that more than 20% of tumor-infiltrating *Cd45^+^* immune cells and *Fsp-1^+^* fibroblasts were positive for human *HSAT2* RNAs (Figure 6, G and H), supporting their transfer from tumor to stromal cells.

We next examined the potential impact of EwS EVs on stromal fibroblasts and myeloid cells, using MRC5 fibroblasts and MUTZ-3 myeloid cells as in vitro models. After the 6 hour exposure to EwS EVs, both MRC5 and MUTZ-3 cells secreted type-I and type-III IFNs (IFN-α, IFN-β and IL-28A/IFN-λ2), with TC32 EVs having the strongest effect (Supplemental Figure 9, A–C). In addition, MUTZ-3 cells secreted proinflammatory and immunosuppressive cytokines, including IL-8, IL-10, IL-20, and IL-35 (Supplemental Figure 9C). Prolonged exposure of at least 72 hours to TC32 EVs increased the number of cells with mitotic defects (Supplemental Figure 9, D and E) and positivity for senescence-associated β-galactosidase (Supplemental Figure 9, F and G), reminiscent of abnormalities caused by ectopic *HSAT2* expression (31). Therefore, *HSAT2,3* RNAs exhibit pathogen-like dinucleotide usage, can be transmitted to stromal immune cells and fibroblasts in the TME, and may contribute to EwS EV-induced proinflammatory responses, mitotic abnormalities, and senescence in adjacent stromal fibroblasts.

### HSAT-enriched EwS EVs activate cGAS and DNA damage pathways in recipient cells.

Despite the highly immunogenic properties, *HSAT2,3* expression did not activate inflammatory responses in EwS cells and tumors (Figure 3G), which could be due to their preferential secretion in EVs and/or their nuclear retention in tumor cells. We also considered a possibility that *HSAT2,3* RNAs in tumor cells may be sequestered in nuclear paraspeckles that are known to capture aberrant RNAs and regulate innate immunity (40, 41). Using paraspeckle *NEAT1* RNA as a positive control, ViewRNA fluorescence ISH (FISH) showed that *HSAT2* RNAs are indeed localized to the nucleus and nuclear envelope in A673, TC32, and A4573 EwS cells, but they are not colocalized with *NEAT1* (Supplemental Figure 10), and thus are not trapped in paraspeckles.

**Figure 7:**
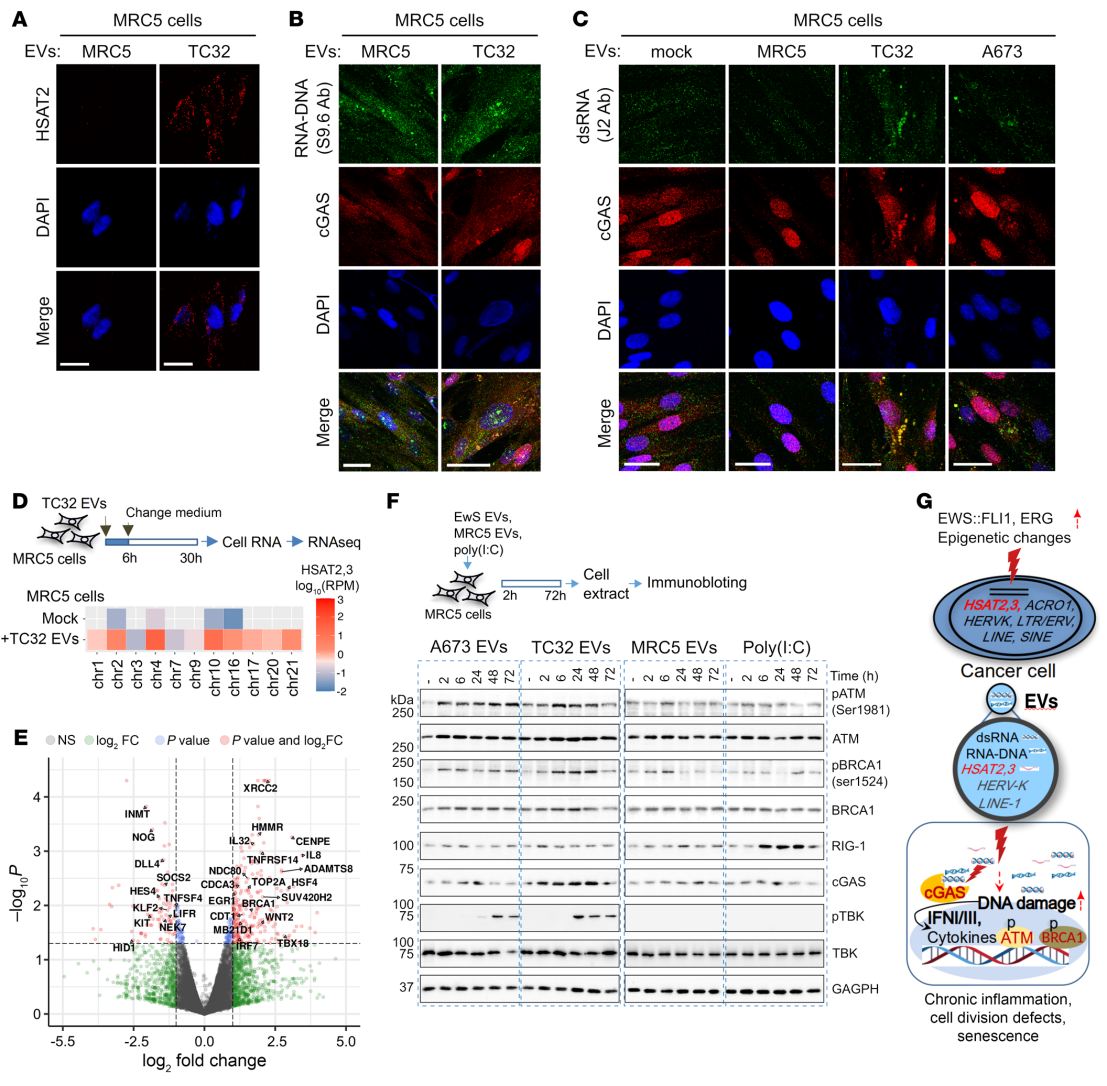
*HSAT* RNA-enriched EwS EVs activate innate immune and DNA damage pathways in the recipient cells. (**A**) ViewRNA-FISH with *HSAT2* probes and (**B** and **C**) immunofluorescence imaging of MRC5 cells treated with TC32 or MRC5 EVs for 24 hours, counterstained with DAPI. Scale bars: 10 μm. (**D**) Experimental outline and chromosomal distribution of *HSAT2,3* RNA-Seq reads from MRC5 cells treated with PBS (mock) or TC32 EVs. Gray color indicates no reads. (**E**) Volcano plot, log_2_ FC versus –log_10_ *P* value of differentially expressed genes in TC32 EV-treated MRC5 cells versus mock. Red dots, genes that passed *P* ≤ 0.05 thresholds and changed >2-fold. (**F**) Immunoblotting pathway analysis of MRC5 cells treated with mock (–), EwS, or MRC5 EVs, or with 5 ng/mL poly (I:C). (**G**) A proposed model of pericentromeric chromatin activation in EwS and PCa, and the effects on stromal cells. Dissemination of *HSAT2,3* and other pathogen-like repeat RNAs in cancer EVs and their accumulation in stromal fibroblasts and immune cells induces DNA damage and cGAS-pTBK1 signaling. Prolonged activation of these pathways due to continuous exposure to cancer EVs, ongoing reverse transcription, or unresolved DNA damage may lead to local and systemic chronic inflammation, mitotic defects, and senescence.

In contrast to tumor cells, *HSAT2* RNAs were detected on the plasma membrane and in the cytosol of MRC5 cells treated with TC32 EVs, but not with MRC5 EVs (Figure 7A). To determine if *HSAT2* and other pathogen-like RNAs in EwS EV-treated cells may engage cytosolic innate immune sensors, we focused on the cyclic GMP-AMP synthase–TANK binding kinase 1 (cGAS-TBK1) pathway known to be activated by pathogen- and self-derived dsDNAs (42, 43), including those of retroelement origins (44). Immunofluorescence microscopy with cGAS and S9.6 (or J2) antibodies revealed large cytosolic granules positive for cGAS, RNA-DNA, and dsRNAs in MRC5 cells treated with TC32 or A673 EwS EVs (Figure 7, B and C), which could be indicative of cGAS activation (45).

To further verify EwS EV-mediated RNA transfer to MRC5 cells and to identify activated pathways, we performed RNA-Seq analysis of TC32 EV-treated and mock control MRC5 cells (Figure 7D, top panel). *HSAT2,3* RNA-Seq reads were detected exclusively in TC32 EV-treated cells and mapped to the 2p11.2, 4p11, 10q11.21, 16q11.2 and 21p11.2 loci (Figure 7D, bottom panel), supporting their EwS cell origin. Also, consistent with the observed secretion of proinflammatory cytokines and IFNs by TC32 EV-treated MRC5 cells (Supplemental Figure 9B), analysis of differentially expressed coding genes in TC32 EV-treated versus mock MRC5 cells revealed upregulation of IFN-I/III and proinflammatory response, including *cGAS/MB21D1*, the IFN regulatory factor 7 (*IRF7*) and proinflammatory cytokines *IL8*, *IL32* and *TNFRSF14* (Figure 7E and Supplemental Table 3). Remarkably however, the largest group of upregulated genes (54 of 297) were those encoding centromere and kinetochore complex components (“*CENPA/NDC80*” module), key regulators of spindle assembly and chromatin condensation (“*PLK1/NEK2*” module), and DNA damage repair proteins (“*BRCA1/TOP2A*” module), as shown by gene ontology (GO) and Reactome network analysis (Supplemental Figure 11 and Supplemental Table 4), supporting links between *HSAT2,3* and cell cycle, kinetochore assembly, DNA damage, and innate immune pathways.

Activation of the above pathways in MRC5 cells treated with A673 or TC32 EVs was further confirmed by immunoblotting. In contrast to MRC5 cells treated with MRC5 EVs or with the dsRNA mimetic poly(I:C), EwS EVs activated DNA damage (pATM and pBRCA1) and innate immune (cGAS and pTBK1) responses, which persisted over the 72 hour time course (Figure 7F). Likewise, compared with EVs from vector control cells, the *HSAT2-*enriched EVs from ERG-expressing prostate cells, especially BPH1, upregulated cGAS, pTBK1, pATM, pBRCA1, and γH2AX levels in MRC5 cells (Supplemental Figure 12). In summary, reactivation of pericentromeric heterochromatin in cancers with aberrantly expressed ETS proteins, including EWS:FLI1 and ERG, results in expression and dissemination of *HSAT2,3* RNAs, which, together with *HERV-K* and other retroelement RNAs present in EVs, may activate DNA damage and cGAS-pTBK innate immune signaling in the recipient immune cells and fibroblasts, driving local and systemic inflammation (Figure 7G).

## Discussion

One of the biggest obstacles for successful immunotherapy in the majority of solid human malignancies is low immunogenicity of the tumor and the proinflammatory immunosuppressive TME (17). Our study demonstrates that reactivation of constitutively silent heterochromatin may reinforce oncogenic gene expression programs and may also contribute to a paradoxical cooccurrence of inflammation and immunosuppression. In particular, we show that elevated expression of *HSAT2,3* RNAs in EwS and PCa as well as in EWS:FLI1-expressing HeLa cells and in ERG-high prostate cells was associated with activation of proinflammatory signaling, DNA damage, G2/M checkpoint, and mitotic spindle/kinetochore maintenance gene expression programs, many of which are known to be dysregulated in both cancers and, in some cases, are directly driven by EWS:FLI1 or ERG (14–16, 46). Moreover, these programs were also activated in EwS EV-treated fibroblasts, contemplating a possibility that *HSAT2,3* RNAs can mediate genotoxic effects of these oncogenic proteins in bystander host cells. The remarkable similarities of gene expression programs in *HSAT2,3*-high cells and tumors could be attributed to the important roles of peri/centromeric RNAs in genome architecture, kinetochore assembly, and mitosis (33, 47, 48). This could also be due to their ability to directly bind and sequester BRCA1 and other proteins that are involved in heterochromatin maintenance, replication, transcription, and splicing (30, 31, 49).

Mechanistically, we show that activation of *HSAT2,3* pericentromeric heterochromatin may occur through the direct binding of EWS:FLI1 (or ERG) to *HSAT3* repeats, primarily those on chromosomes 4p11 and 10q11.21, and less so, on 2p11.2 and 21p11.2. While the exact mechanism remains to be established, preferential binding to *HSAT3* repeats could be due to their enrichment with consecutively repeated GGAAT motifs, which are high-affinity binding sites for both EWS:FLI1 and ERG in a complex with EWS (5, 6, 12, 13). Binding to *HSAT3* may then activate the adjacent *HSAT2* repeats, given their proximity in certain genomic locations, particularly on chromosomes 2p11.2 and 10q11.21. It cannot be excluded, however, that *HSAT2* repeats can be directly activated by overexpressed ERG in certain cellular contexts, as hinted by its more promiscuous consensus binding motifs and by upregulation of *HSAT2* (but not *HSAT3*) in ERG-high BPH1, PNT1, and RWPE1 nontransformed prostate cells.

Our work also shows that EwS and PCa tumor cells have developed the ability to release *HSAT2,3* RNAs in EVs. A potential mechanism may involve their sorting in EVs in a form of dsRNAs and RNA-DNA hybrids, and retention of ssRNAs in nuclei of tumor cells. This could also explain a lack of immune response in *HSAT2,3*-expressing EwS cells compared with EwS EV-treated cells, where cytosolic accumulation of *HSAT2* RNAs was associated with vigorous IFN-I/III and proinflammatory responses, activation of cGAS-pTBK1 signaling, and a buildup of cytosolic RNA-DNA and dsRNA-positive cGAS aggregates. The cGAS-TBK1 innate immune pathway is emerging as a key driver of cancer-associated chronic inflammation and immunosuppression (42, 50). Upon engagement with cytosolic DNA, cGAS forms liquid phase droplets, where it becomes activated (43, 45). cGAS can also bind to RNA-DNA hybrids including retroelements (44), and can restrict RNA virus infections (51). It is plausible that similar mechanisms may operate in the host cells, upon exposure to cancer EVs, as a result of cytosolic accumulation of *HSAT2,3* and retroelement RNAs, some of which may be able to reverse transcribe (38, 52, 53). Alternatively, cGAS activation in cancer EV-treated cells can be driven by DNA damage and leakage of genomic DNA to the cytosol, which may also lead to senescence, as observed in this study and by others (42, 43).

In other cancers and senescence-associated disorders, aberrant activation of pericentromeric chromatin has been linked to *BRCA1* mutations (49, 54) and malfunction of various epigenetic modifiers, including histone deacetylase SIRT6 (55) and DNA methyltransferases DNMT1 and DMNT3B (56). *HSAT2* expression was observed in colorectal, pancreatic, and ovarian carcinomas, and was linked to immunosuppression (25, 57). It is thus tempting to speculate that pericentromeric chromatin activation is a convergent point for multiple oncogenic drivers in diverse human malignancies. Our current and previous (18, 19) studies indicate that this process may also extend to normal host cells in the TME and systemic circulation, causing DNA damage, proinflammatory responses, senescence, and immunosuppressive phenotypes (Figure 7G). In this regard, our investigation argues against the proposed strategy to enhance tumor immunogenicity by inducing expression of *ERVs*, *LINEs*, and other repeat RNAs (58), as this state of so-called viral mimicry may ultimately lead to chronic inflammation, immunosuppression, and senescence. Quite the contrary, in order to reactivate the immune system, further investigation should be focused on developing containment strategies, to prevent their expression in tumor cells, dissemination in EVs, and infection of target cells.

## Methods

### Sex as a biological variable.

The EwS patient cohorts included 18 males and 20 females. Sex was not considered as a biological variable in this study; similar results were obtained for both sexes. Relevant clinical information (age, sex, tumor site, and stage) is shown in Supplemental Table 2.

### Plasma EV isolation.

Plasma specimens were kept at –80°C. After thawing, plasma (approximately 1–2 mL/patient) was cleared by 2-step centrifugation at 2,000*g* for 10 minutes and at 10,000*g* for 20 minutes, to remove cell debris and aggregates. Supernatants were diluted 1:4 with ice-cold PBS, passed through 0.45 μm filters and centrifuged for 90 minutes at 4°C at 100,000*g* (S110-AT rotor, Thermo Fisher Scientific). To ensure removal of plasma contaminants, EV-containing pellets were resuspended in 3 mL of PBS and pulled down at 100,000*g* for 60 minutes. Pellets were dissolved in PBS and examined using the nanoparticle tracking system (NS300 NTA).

### Cell lines and EV isolation.

Cell lines and growth conditions are listed in Supplemental Table 1. For EV isolation, cells were cultured on 15 cm plates (10 × 10^6^ cells/plate) in growth medium containing 2% EV-depleted FBS (System Biosciences) for 18–24 hours. Conditioned medium (CM) from the same number of cells was collected and cleared by sequential centrifugation at 2,000*g* for 10 minutes and 10,000*g* for 20 minutes, passed through 0.45 μm filters, and concentrated on Amicon Ultra-15 mL-30 K tubes (Sigma-Aldrich). Concentrated CM was then diluted 1:1 with PBS and centrifuged at 100,000*g* for 90 minutes. Crude EVs were resuspended in PBS and pelleted again at 100,000*g* for 60 minutes. EV pellets were either directly used for RNA isolation with the mirVana RNA kit (AM1561, Thermo Fisher Scientific),or resuspended in PBS for downstream analyses.

### RNA immunoprecipitation from the conditioned medium.

CM was precleared by filtering and sequential low-speed centrifugation, concentrated on Amicon Ultra tubes, and supplemented with the EV lysis buffer (10 mM Tris-HCl, pH 8.0, 1 mM EDTA, 0.5% Triton X-100, 0.1% SDS [all from Sigma-Aldrich]) in a total volume of 2 mL. The CM was then precleared with protein G magnetic beads (S1430S, New England Biolabs) at 4°C for 1 hour, and incubated for 3 hours with 5 μL of S9.6 (ENH001, Kerafast) or J2 (ES2001) antibodies, or normal mouse IgG (sc-2025, Santa Cruz) immobilized on 30 μL of protein G magnetic beads. After removal of unbound fractions, magnetic beads were washed with the 140 mM NaCl EV lysis buffer, and bound RNAs were eluted with 2 μg/mL proteinase K (19131, Qiagen) and isolated with the mirVana kit (AM1561, Thermo Fisher Scientific) using 1 ng of *MS2* RNA (10165948001, Sigma-Aldrich) as a spike-in control.

### Reverse transcription-PCR.

Purified RNAs were treated with Turbo DNase (AM1907, Thermo Fisher Scientific) and quantified using the Qubit RNA high sensitivity kit (Q32852, Thermo Fisher Scientific). RNA integrity was analyzed using the Agilent RNA Pico 6000 kit on a 2100 Bioanalyzer instrument (Agilent Technologies). cDNA was synthesized using SuperScript III Reverse Transcriptase (18080044, Thermo Fisher Scientific), 100–500 ng of DNase-treated RNA and random hexamer primers. Quantitative PCR (qPCR) was performed on the ViiA 7 Real-Time PCR instrument (Thermo Fisher Scientific) using the PowerUp SYBR Green Master Mix (A25777, Thermo Fisher Scientific) and the respective primer sets (Supplemental Table 5). RT-negative controls were included in all qPCR reactions. Expression of target transcripts was quantified by ΔΔCt method using normalized Ct values. qPCR products were analyzed by 1.5% agarose gel electrophoresis and confirmed by Sanger sequencing. Droplet-based digital PCR (ddPCR) was performed on the QX100 Droplet Digital PCR system (Bio-Rad) using the QX200 ddPCR EvaGreen Supermix (1864033, Bio-Rad). PCR reactions were incubated at 95°C for 5 minutes, followed by 40 cycles of 95°C for 30 seconds and 58°C for 1 minute, with final incubations at 4°C for 5 minutes and at 90°C for 5 minutes. The average number of accepted droplets for the valid measurement results was approximately 17,000.

### HeLa and VCaP cell culture assays.

Transient transfection of HeLa cells was performed on 6-well plates (0.5 × 10^6^ cells/well) using the Lipofectamine 2000 reagent and the p3xFLAG-CMV-10-EWS:FLI1 plasmid (59), a gift from Beat Schäfer (University Children’s Hospital, Zurich, Switzerland). Cells were collected at 24, 48, and 72 hours after transfection and analyzed by immunoblotting and RNA-Seq. VCaP cells were grown in phenol red–free DMEM supplemented with 10% charcoal stripped FBS (both from Thermo Fisher Scientific) for 24 hours before stimulation with 1 nM R1881/methyltrienolone (Sigma-Aldrich) for 48 hours. Where indicated, cells were transfected with On-target plus SMART pool siRNAs (Dharmacon), targeting human ERG (L-003886-00-0005) or nontargeting control (Src: d-001810-01-05) using DharmaFECT transfection reagent (Dharmacon). Cells and the respective CM were collected 48 hours after treatment with R1881 and siRNAs and were used for immunoblotting and RNA isolation.

### Immunoblotting.

Whole cell extracts and EVs were prepared in lysis buffer (50 mM NaCl, 10 mM Tris-HCl, pH 7.8, 1 mM DTT, 0.25% NP-40) supplemented with complete protease inhibitor cocktail (Roche Diagnostics), resolved on SDS-10% polyacrylamide gel (10–25 μg protein/lane) and transferred onto nitrocellulose membrane (0.45 μm; 162-0115, Bio-Rad). The membrane was incubated for 30 minutes in 5% nonfat dry milk/TBST (150 mM NaCl, 20 mM Tris-HCl, pH 7.8, 0.1% Tween-20) blocking buffer followed by overnight incubation at 4°C with primary antibodies listed in Supplemental Table 6. The membranes were washed with TBST, incubated with horseradish peroxidase-conjugated secondary antibodies at 1:5,000 dilution for 40 minutes at room temperature, washed with TBST, developed with ECL reagents, and documented by digital imaging using ChemiDoc MP Imaging system (Bio-Rad).

### Bio-Plex proinflammatory cytokine profiling.

MRC5 fibroblasts or MUTZ-3 myeloid cells were treated for 6 hours with 25–50 μL of EVs (2.5–5 × 10^9^ particles/50,000 cells/well in 24-well plates) or mock control (PBS). The medium was replaced with the fresh medium containing 2% FBS, and cells were grown for another 18 hours. The CM was collected and analyzed using the magnetic bead-based multiplex immunoassay Bio-Plex 200 system and the 37-plex human inflammation panel (171AL001M, Bio-Rad). Standard and sample cytokine concentrations were calculated using the Bio-Rad Bio-Plex 200 software. Background-corrected signals for each cytokine were normalized to the mean signal intensity obtained for each sample and were plotted using the mean and SD from 3 biological replicates.

### Senescence-associated β-galactosidase staining.

MRC5 cells were plated in 24-well plates at low confluency (25,000 cells/well), treated with 50 μL of EVs (5 × 10^9^ particles) or PBS in 500 μL of 2% FBS medium for 24 hours and grown for 3–5 days in a fresh 10% FBS medium. Senescent cells were detected with the senescence β-gal staining kit (9860, Cell Signaling).

### Immunofluorescence microscopy and ViewRNA-FISH.

MRC5 and EwS cells were plated on coverslips in 24-well plates and treated with EVs (approximately 5 × 10^9^ particles/50,000 cells/well). For immunofluorescence (IF), cells were fixed with 4% paraformaldehyde, permeabilized with 0.1% Triton X-100, and blocked with 5% BSA for 1 hour. Staining was done using α-Tubulin (3873, Cell Signaling) and Aurora A (14475, Cell Signaling) antibodies (Supplemental Table 6) overnight at 4°C, followed by 1 hour incubation with Alexa Fluor-488 and -594 conjugated anti-mouse (Thermo Fisher Scientific, A-11001) and anti-rabbit (Thermo Fisher Scientific, A-11012) secondary antibodies. ViewRNA-FISH was done using the ViewRNA Cell Plus Assay Kit (88-19000-99, Thermo Fisher Scientific) and probe sets for *HSAT2* (VA6-19493-06) and *NEAT1* (VA4-20324). Coverslips were mounted with ProlongGold antifade reagent with DAPI (P36935, Invitrogen). Images were acquired using a Zeiss confocal microscope with ×63 oil immersion lens.

### ViewRNA ISH.

Archived formalin-fixed paraffin-embedded (FFPE) sections of EwS tumors were provided by Katja Specht and Katja Steiger (Institute of Pathology, Technical University of Munich School of Medicine, Munich, Germany). FFPE sections of mouse xenografts of TC32 EwS cells were from a previous study (39), provided by Amal El-Naggar (University of British Columbia, Vancouver). ViewRNA-ISH was performed using the tissue 2-Plex assay kit (QVT0012, Thermo Fisher Scientific) and probe against human *HERV-Kpol* (VF1-18759-06) and *HSAT2* (VA6-19493-06), and mouse *S100a4/Fsp-1* (VB1-18990-06) and *Ptprc/Cd45* (VB1-12657-06) RNAs. Briefly, 5 μm FFPE sections were baked and deparaffinized, followed by heat pretreatment and protease digestion. Target probes were then hybridized for 2 hours at 40°C followed by ViewRNA signal amplification. Signal detection was performed sequentially, using Fast Blue and Fast Red substrates, and slides were then counterstained with hematoxylin. Signal was considered specific if normal kidney tissue and no-probe controls showed less than 1 dot per 20 cells. Regions of interest were captured at ×100 magnification using a Leica DM2000 microscope and LAS software with a pixel resolution of 0.04 μm/pixel. Stain separation of the fast blue, fast red, and nonspecific background signals were achieved using a python implementation of the independent component analysis approach (60). The resulting deconvoluted images were analyzed using a custom rule set built in the object-oriented machine learning software Developer XD (Definiens AG, Munich, Germany). The rule set first separated nuclear regions from nonspecific signal using a weighted combination of all isolated stain intensities, with watershed and object reshaping algorithms being employed to identify and separate closely spaced nuclei. Blue and red spots were then detected with the fast blue and fast red channels, respectively. The resulting cell objects were exported as a table listing the number and type of spots within each cell. The number of cells containing at least 1 red spot, 1 blue spot, or both were tabulated as a fraction of total cells detected.

### Whole transcriptome RNA-Seq.

Total cellular RNAs from EwS cell lines (100–500 ng) and EV RNAs (20–200 ng) were depleted of ribosomal RNA using the RiboZero rRNA removal kit (20020596, Illumina) and used to construct strand-specific RNA-Seq libraries with the TruSeq Stranded Total RNA library prep. The quality of libraries was confirmed using the Agilent High Sensitivity DNA assay, and those of sufficient quality and quantity were pooled in sets of 4 and sequenced using the HiSeq PE cluster kit v4 on the Illumina HiSeq 2500 sequencer, generating, on average, approximately 30–100 million paired-end 100-bp reads/sample. RNA-Seq libraries from HeLa and VCaP cells were prepared using the KAPA RNA HyperPrep Kit with RiboErase (HMR) (8098140702, Roche) and sequenced to about 100 million paired-end 150 bp reads/sample on an Illumina NovaSeq sequencer.

### RNA-Seq data analysis of repeat transcripts.

Paired-end strand-specific 100 or 125 bp reads were aligned to human genome build 37 (hg19/GRCh37) using the Burrows-Wheeler Aligner (BWA; v.0.7.13) with subsequent filtering of ribosomal and tRNA reads, followed by removal of secondary alignments and reads with mapping score less than 30. Transcript expression was quantified as the number of reads per million (RPM). Based on hg19-compliant RepeatMasker annotations (https://www.repeatmasker.org), reads were assigned to different repeat classes using bedtools suite 2.29.2. *HSAT3* was defined by simple GAATG/CATTC repeats larger than 1 kb and located in pericentromeric regions. To quantify expression of different repeat classes, overlapping annotations were resolved, giving priority to features in a hierarchical order: miRNA > piRNA > lncRNA/lincRNA > RepeatMasker annotations. The annotated reads were partitioned into 2 large groups: nonrepeat transcripts, represented by protein coding (including 5′- and 3′-UTRs and introns) and noncoding (pseudogenes, snRNAs, miRNAs, and lncRNAs) and repeat transcripts. The latter group was further subdivided into *SINEs*, *LINEs*, DNA and *LTR/ERVs,* low complexity, simple (microsatellite), and satellite (pericentromeric and centromeric repeats). The results were plotted using the R statistical environment (https://www.r-project.org). Plots were generated using the ggplot2 package.

### Coding gene alignments, differential expression and GSEA.

For protein coding genes, reads were aligned to the human genome (release 87 for GRCh37) with STAR v2.6.0c (61). Counts for genes were derived with the SUBREAD suite (2.0.6) featureCounts tool (62) using GENCODE annotations (release 19). Differential expression analysis of protein coding genes was performed with edgeR 3.42.4 tools (63). Transcripts showing 2-fold difference and *P* value < 0.05 were considered as differentially expressed. For GSEA, RNA-Seq read counts were normalized with DESeq2 (64) followed by calculation of enrichment score using the GSEA v.4.2.3 and Molecular Signature Database (MSigDB) version 7.5.1 (http://www.gsea-msigdb.org/gsea/msigdb/collections.jsp). Normalized enrichment score (NES) was used as a measure of coordinated changes in hallmark gene pathways with “Gene Set” as the permutation type. The permutation number was set at 1,000, FDR values of less than 0.25 (or normalized *P* value < 0.01 for pathways that did not pass FDR threshold) were considered significant. Leading edge genes were identified for each significantly enriched pathway, and their contribution to the signal was assessed and visualized by *Z*-score scaling. Heatmaps were generated using the pheatmap R statistical programming package.

### GO and reactome functional interaction network analysis.

GO analysis and functional enrichment in the molecular function, biological process, and cellular component categories were performed using GOrilla application (65). Highly enriched “Chromatin-remodeling”, “Cellular response to DNA damage”, and “Cell division” GO categories were further analyzed using the Reactome (https://reactome.org) database V71 and Reactome FIViz 2019 Cytoscape plugin (66), to identify network modules of highly connected genes and their significantly enriched (FDR-adjusted *P* value < 0.05) biological processes.

### ChIP-Seq data analysis.

Publicly available ERG ChIP-Seq data sets (GSE28950) (9) were extracted as FASTQ files using Gene Expression Omnibus tools. The EWS:FLI1 ChIP-Seq data (46) were retrieved from the BAM files using Picard v.2.9.0 and converted to FASTQ files. The reads were mapped to hg19 (GRCh37.p13) reference assembly using Bowtie2 aligner (v.2.3.4.1). Peaks were called using MACS2 (v2.2.6) default settings, with Q ≤ 0.05 as the threshold. Unmapped reads were assembled into contigs using Trinity v.2.6.6. Quality of the assembled contigs was assessed with utility scripts from trinity suite and limited to the size of 700 bases or fewer. To establish their identities and locations, the contigs were aligned against the 24-mer *HSAT2,3* motif reference database (26) and RepeatMasker-annotated satellite repeats using BLAST tools.

### Data availability.

RNA-Seq data sets generated from plasma EVs of 12 patients with EwS and 7 healthy donors, cell lines, and their EVs were deposited at the Sequence Read Archive (SRA) under the accession number PRJNA548159. Values for all data points in graphs are reported in the Supporting Data Values file.

### Statistics.

All statistical analyses were conducted using R statistical environment v4.3.2 (https://www.R-project.org/). Additional packages used for data analysis and visualization included Bioconductor (https://www.bioconductor.org) and CRAN (https://cran.r-project.org/web/packages/). Normal distribution of data in RNA-Seq and ddPCR data sets was confirmed with Shapiro-Wilk test. To correct for multiple comparisons, the unpaired *t* test on log-transformed values were adjusted using Benjamini-Hochberg (BH) test. For data that failed a normality test, the Wilcoxon signed-rank test was applied. For comparisons among more than 2 groups of samples, 1-way ANOVA or Welch F test (in cases when homoscedasticity condition was violated) were applied. To check for associations between biochemical recurrence (BCR) rates and *HSAT2,3* expression, survival analysis was performed using the R Survival package (v3.5–7) with 0.1 as cutoff. Cox proportional hazards model was then fit using patients groups divided by median *HSAT2,3* abundance using BCR as endpoint. Data visualization was performed using the survminer R package (0.4.9).

### Study approval.

Blood plasma specimens were obtained from patients with EwS who were enrolled in Genewing (the EW cohort; Institute Curie, Paris, France) and NEO-IDENT (the TUM cohort; the Technical University of Munich, Munich, Germany) studies, and from age-matching healthy donors undergoing routine checkups at the Technical University of Munich (Munich, Germany). Each cohort received the approvals from the respective institutional review boards, Comité de Protection des Personnes, Ile-deFrance (09-12037), and the Ethikkommission der Medizinischen Fakultät der Technischen Universität München (2562/09 and 649/20S). Protocols for clinical sample handling and processing were reviewed and approved by the University of Toronto (35129 and 36089).

## Author contributions

PR provided computational and statistical analysis, data interpretation and figure preparation. VE designed the study, performed experiments and wrote the manuscript. MCP, ZZ, and HG purified EVs and performed RT-ddPCR and immunoblotting. AM performed immunofluorescence microscopy. SL contributed to transfection experiments. HG, UT, EL, OD, and SEGB provided plasma samples and interpreted the data. MOM performed reactome data analysis. SHZ and VP provided intellectual and technical support. MS and MEC provided VCaP and ERG-expressing prostate cells, conducted experiments, and interpreted the data. TDM and MZ conducted quantitative image spot analysis. LEH, JDM, and LDS supervised RNA-Seq and data analysis. LR, LDS, MEC, SEGB, and PHS secured funding and reviewed and edited the manuscript. All authors reviewed and approved the manuscript. The first co–authorship is shared between PR and VE based on their equal contribution and mutual agreement.

## Supplementary Material

Supplemental data

Unedited blot and gel images

Supplemental tables 1-5

Supporting data values

## Version 1 03/26/2024

In-Press Preview

## Version 2 05/01/2024

Electronic publication
