# Supplementary material for: Oncogenic ETS fusions promote DNA damage and proinflammatory responses via pericentromeric RNAs in extracellular vesicles
Source: J Clin Invest. 2024 Mar 26;134(9):e169470. doi: 10.1172/JCI169470 (PMC11060741; doi:10.1172/JCI169470)
Supplement: Supplemental data [file jci-134-169470-s078.pdf]

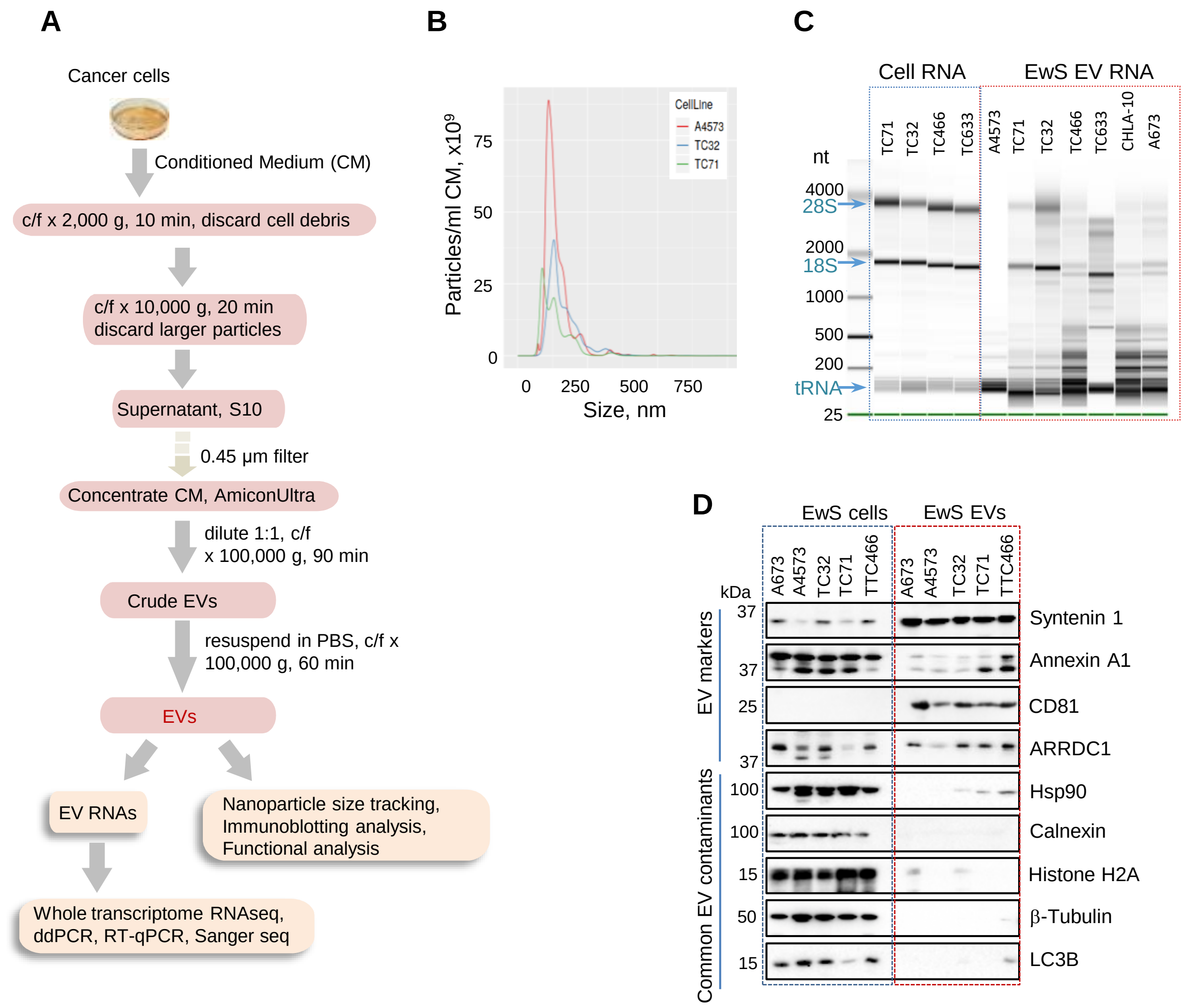

Supplemental Figure 1. Purification and characterization of EV preparations from EwS cell lines. (A) Outline of the EV purification protocol. **(B)** Nanoparticle size tracking analysis. **(C)** Bioanalyzer electropherogram of cellular and EV RNAs using the Agilent Pico RNA chip. Arrows indicate the major bands of 18S and 28S ribosomal RNAs and tRNAs. **(D)** Immunoblotting analysis of EwS cells and the respective EVs. Representative data of three independent experiments is shown in **B-D**.

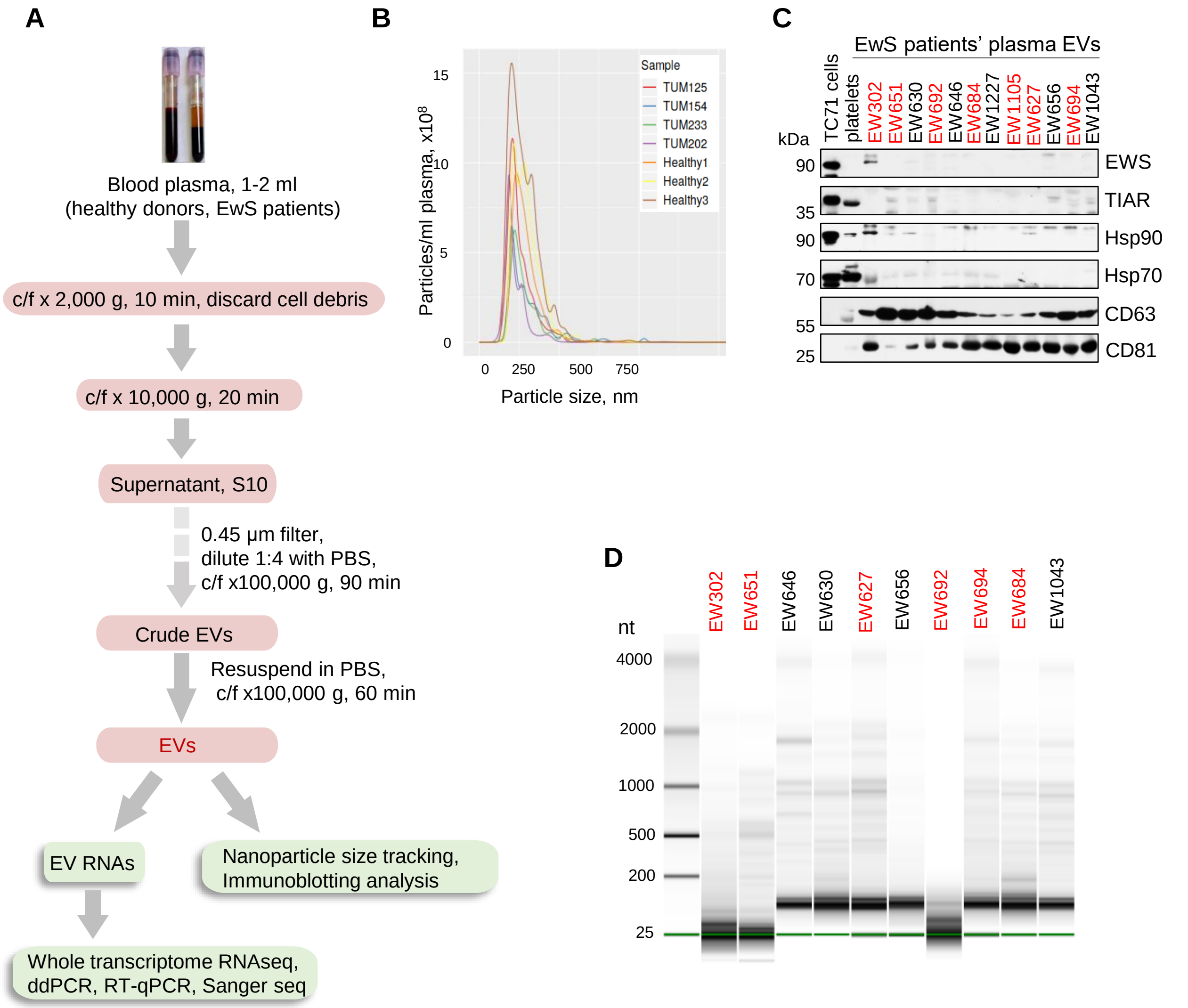

Supplemental Figure 2. Purification and characterization of plasma EVs from healthy donors and EwS patients. **(A)** Outline of plasma EV purification protocol. **(B, C)** Nanoparticle size tracking analysis **B** and immunoblotting **C** of plasma EVs from of healthy donors and EwS patients with localized (black) or metastatic (red) disease. TC71 cells and human platelets are shown here for comparisons. (**D)** Bioanalyzer electropherogram of plasma EV RNAs using the Agilent Pico RNA chip. Representative data from at least three independent preparations are shown.

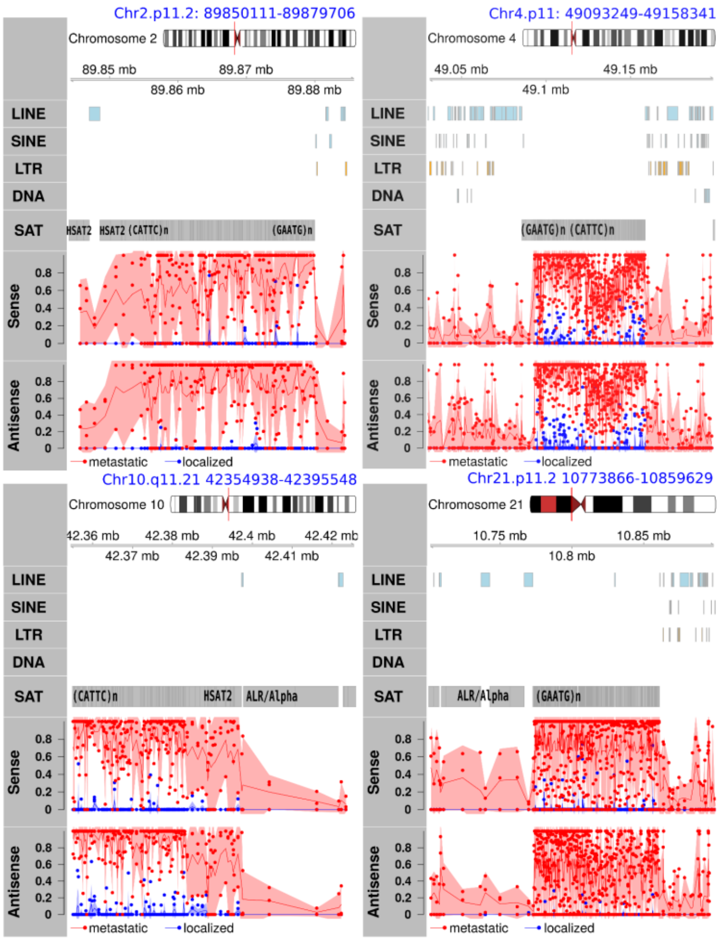

Supplemental Figure 3. Genomic origins of *HSAT2,3* RNAs detected in plasma EVs of EwS patients. Chromosomal distribution of uniquely mapped *HSAT2,3* RNAseq reads detected in plasma of patients with localized (blue dots) or metastatic (red dots) disease. Sense and antisense strand coverage of four major genomic loci is shown.

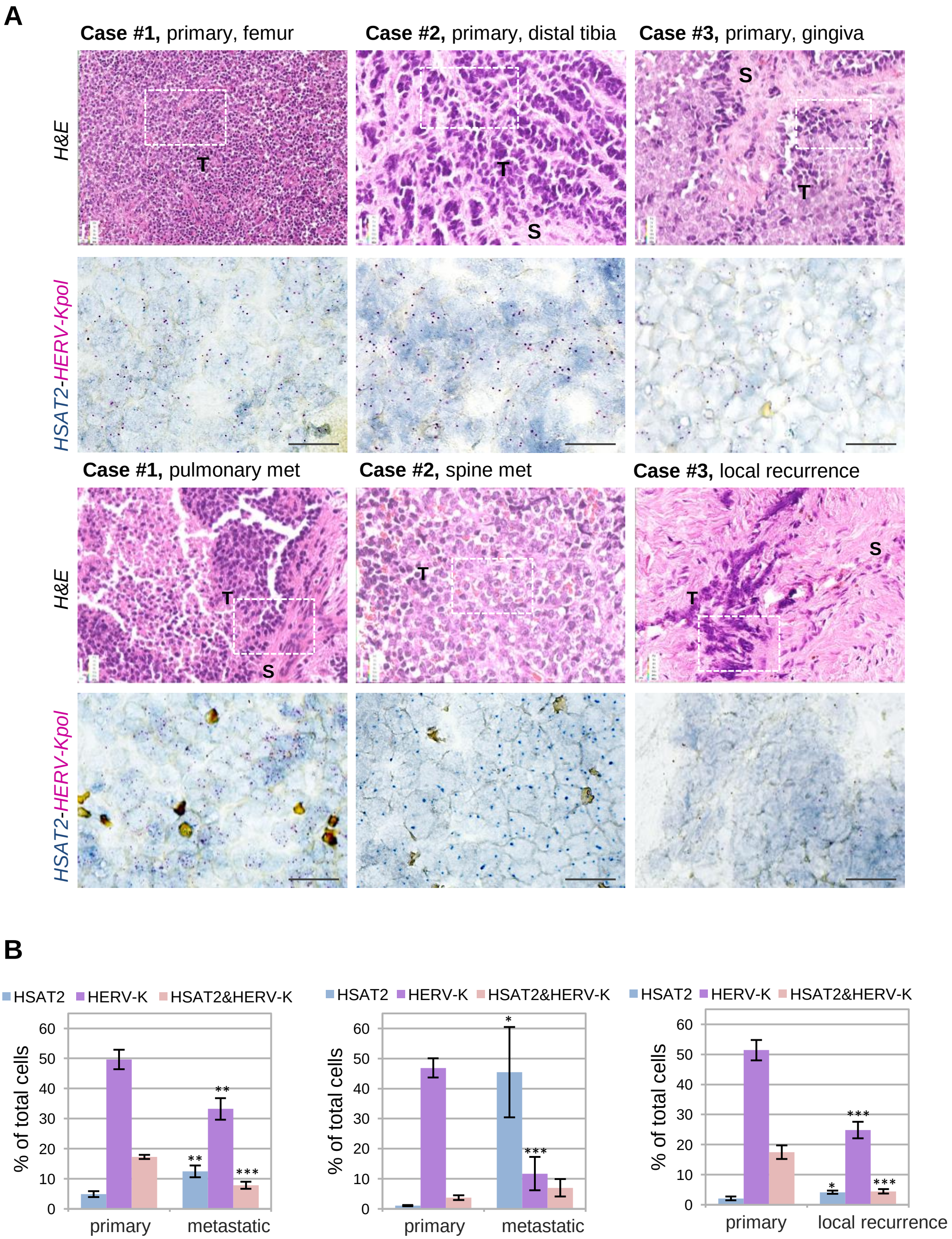

Supplemental Figure 4. *HSAT2* and *HERV-K* expression in matching primary and metastatic tumors from three EwS patients. (A) Representative images of hematoxylin and eosin (H&E) staining (top panels; x40 magnification) and detection of *HSAT2* and *HERV-K* RNAs by ViewRNA-ISH (bottom panels; x100 magnification), counterstained with hematoxylin. ViewRNA images were taken from the areas denoted by squares in the respective top panels. T- tumor, S- stroma. Scale bars, 20 µm. **(B)** Quantification of images shown in **A** using the Developer XD machine learning software. Percentage of cells positive for *HSAT2*, *HERV-K* or both is shown. Data are expressed as mean ± SD. Comparisons made relative to primary tumor; unpaired t-tests with BH adjustment,*$p<0.05$, **$p<0.005$, ***$p<0.0005$.

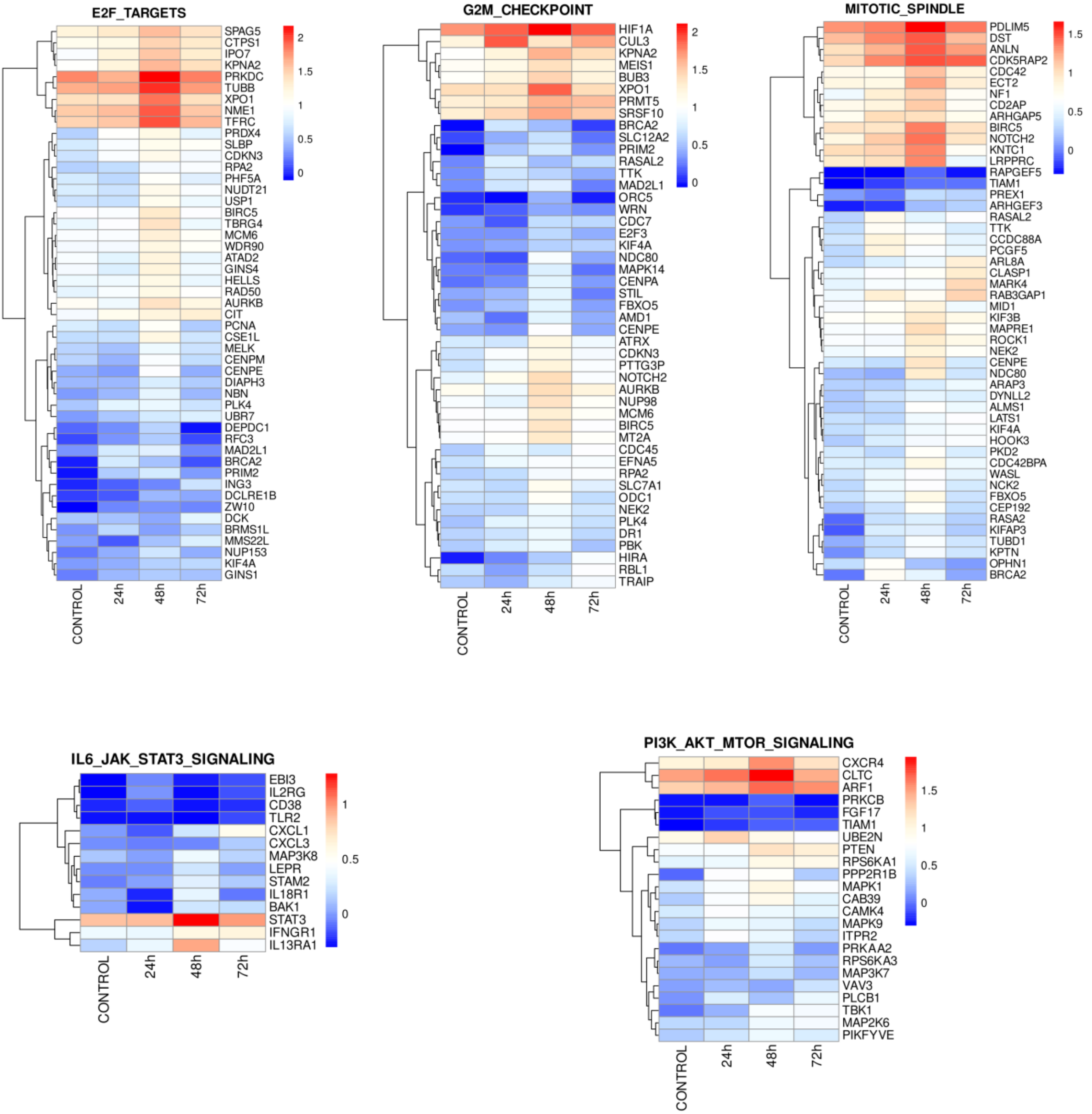

Supplemental Figure 5. Activation of cell cycle, proliferation and proinflammatory pathways in EWS::FLI1 expressing HeLa cells. Hallmark pathways activated in HeLa cells transiently expressing EWS::FLI1 over the 24-72h time course, or vector control (at 72h). Columns represent average expression values of normalized RNAseq reads, two replicates per condition. Core enriched genes identified by the GSEA software are shown.

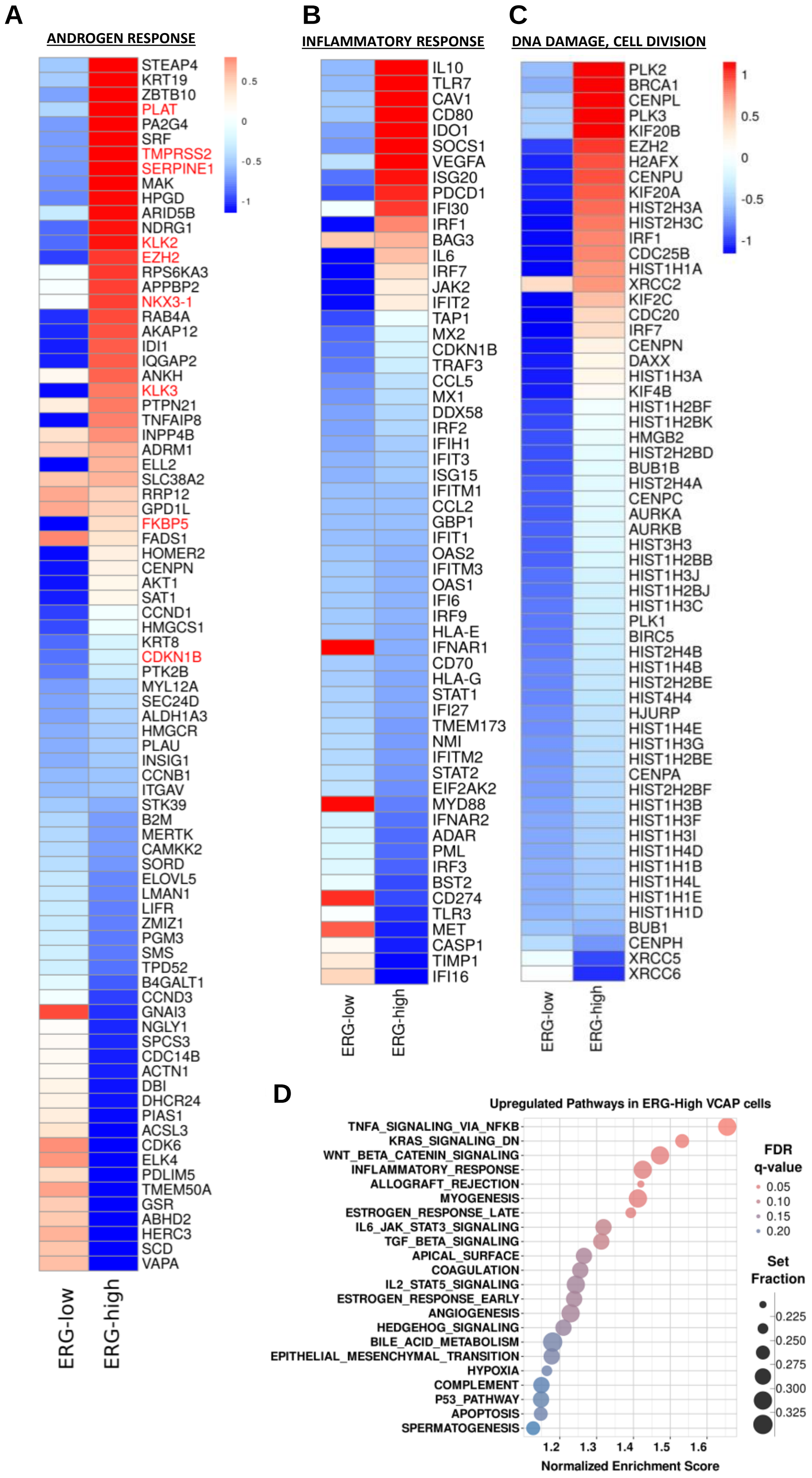

Supplemental Figure 6. *HSAT2,3* upregulation in ERG-high VCaP cells is linked to proinflammatory and DNA damage pathways.
(A-C) Hallmark pathways activated in ERG-low and ERG-high VCaP cells identified using customized hallmark gene sets from “Androgen response”, “IFN response”, “Inflammatory response”, “Nucleosome assembly”, “Chromatin silencing” and “CENP-A nucleosome assembly”. Columns represent average expression values of normalized RNAseq reads, two replicates per condition. **(D)** GSEA analysis of enriched pathways in ERG-high vs ERG-low VCaP cells. Circle size indicates gene set size, and circle color indicates the FDR adjusted q value.

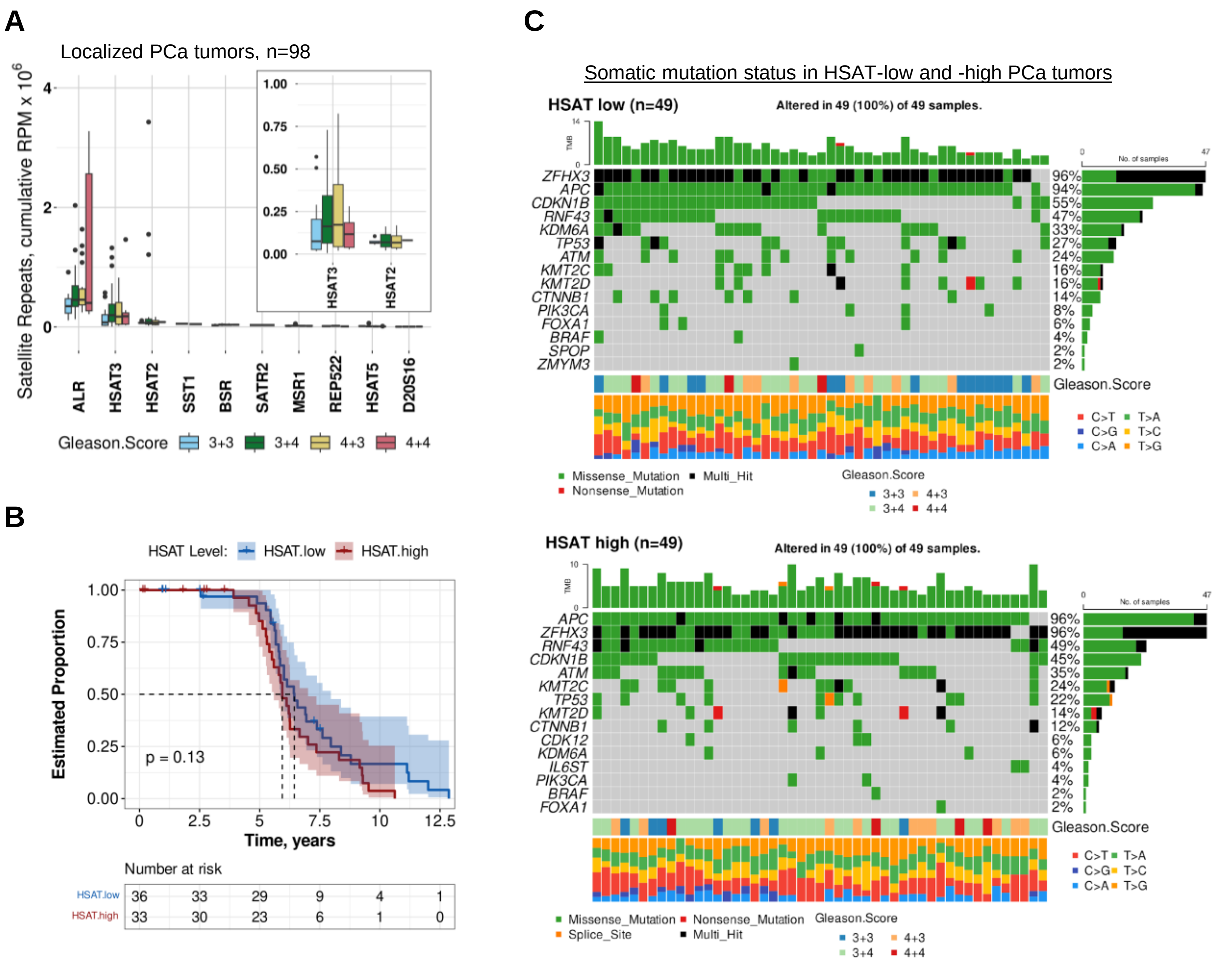

Supplemental Figure 7. High *HSAT2,3* expression in localized PCa tumors is not associated with accumulation of specific somatic mutations, but may be linked to slightly increased biochemical recurrence and tumor grade.
(A) Expression of RepeatMasker-annotated satellite transcripts in localized PCa tumors (n=98) with Gleason score 6-8, shown as reads per million mapped reads (RPM). *HSAT2* and *HSAT3* read counts are also shown separately in the insert. Data are mean RPM ± SEM. (**B**) Kaplan-Meier curve for patients with *HSAT2,3*-high and -low levels. The number of patients for each time point is indicated in the table below. A Cox proportional hazards model was fit for the respective cohorts of patients, with biochemical recurrence as the endpoint. (**C**) Detection of somatic mutations in *HSAT3*-high and -low tumors stratified based on median *HSAT3* RNA expression.

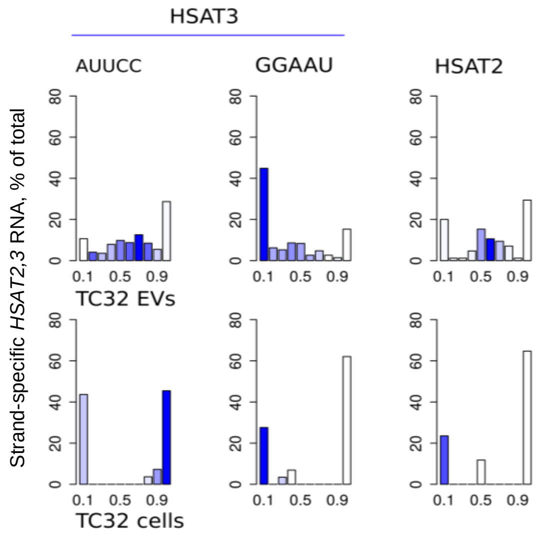

Supplemental Figure 8. Double-strand *HSAT2,3* RNAs are present in EVs but not in tumor cells. Strand-specific alignment of sense and antisense *HSAT2,3* reads mapped to the same locus are shown. The Y-axis, percentage of reads in sense or antisense orientation relative to the reference genome; the X-axis, a Jaccard coefficient calculated as antisense/total reads. Values close to 0.5 indicate an equal proportion of sense and antisense transcripts, while ratios close to zero or 1 represent transcripts in either sense or antisense orientation. Read abundance in each bin is reflected by color coding, with dark blue indicating higher coverage.

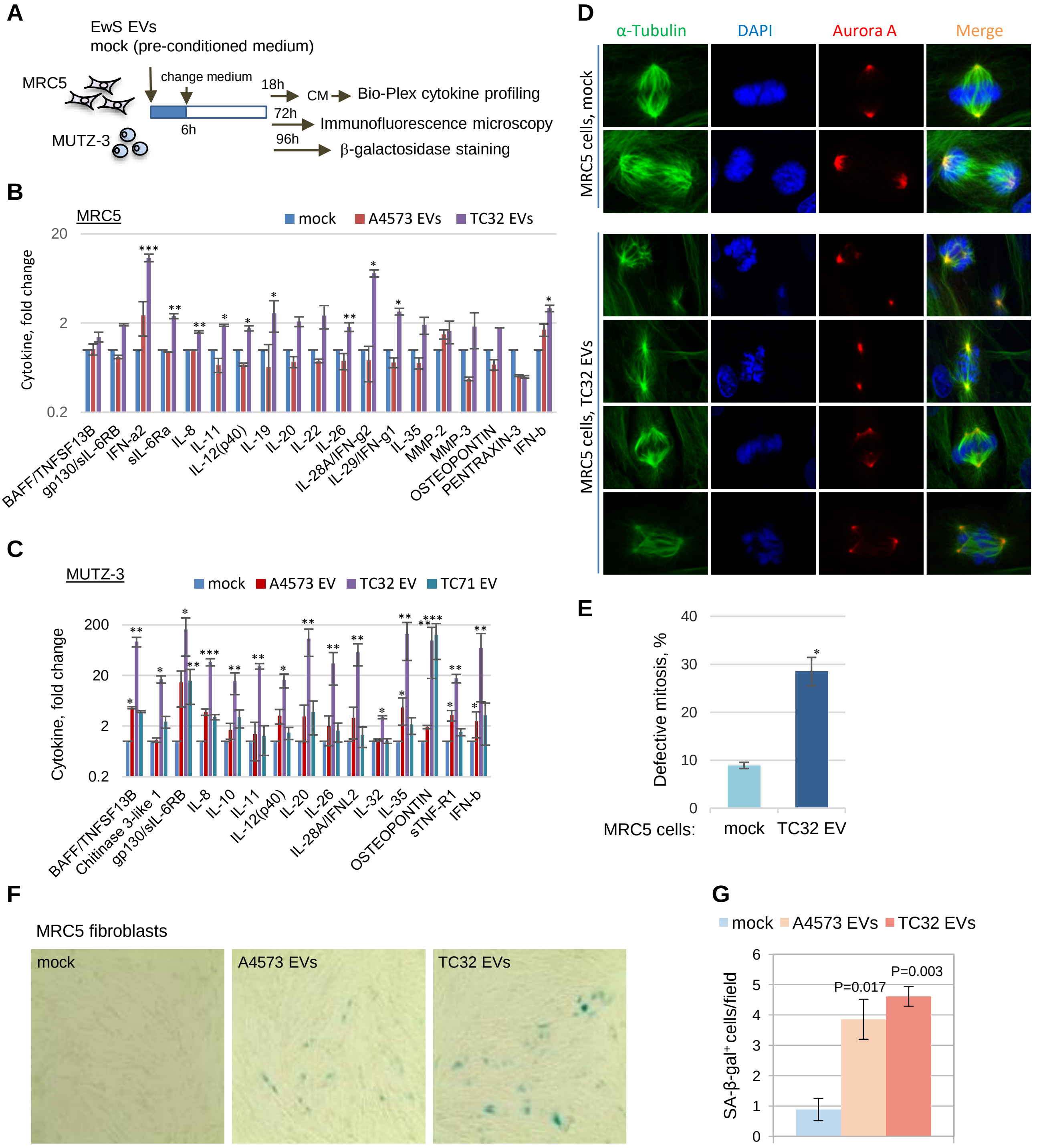

Supplemental Figure 9. EwS EVs induce proinflammatory responses, mitotic defects and senescence in target cells. **(A)** Outline of treatment experiments. **(B, C)** Bio-Plex proinflammatory cytokine profiling of CM from mock and EwS EV-treated MRC5 and MUTZ-3 cells, from 2 and 3 biological replicates, respectively. Data are mean ± SD. Fold change relative to mock (set as 1 for each cytokine), *p<0.05, **p≤0.01; ***p<0.005, paired two-tailed t-test. **(D)** Immunofluorescence imaging of MRC5 cells treated with TC32 EVs or mock, counterstained with DAPI. Original magnification, x100. Note that treatment with TC32 EVs did not affect Aurora A localization to the poles or bipolar α-tubulin-positive mitotic spindle assembly, but increased a number of cells with monopolar, multipolar and disorganized mitotic spindles. **(E)** Data quantification from **D**. Comparisons relative to mock; *p<0.05, unpaired two-tailed t-test. **(F)** Accumulation of senescence-associated (SA) β-galactosidase in mock or EwS EV-treated MRC5 fibroblasts. Original magnification, x40. **(G)** Data quantification from **F**. Comparisons relative to mock; p-values, unpaired two-tailed t-test with BH adjustment.

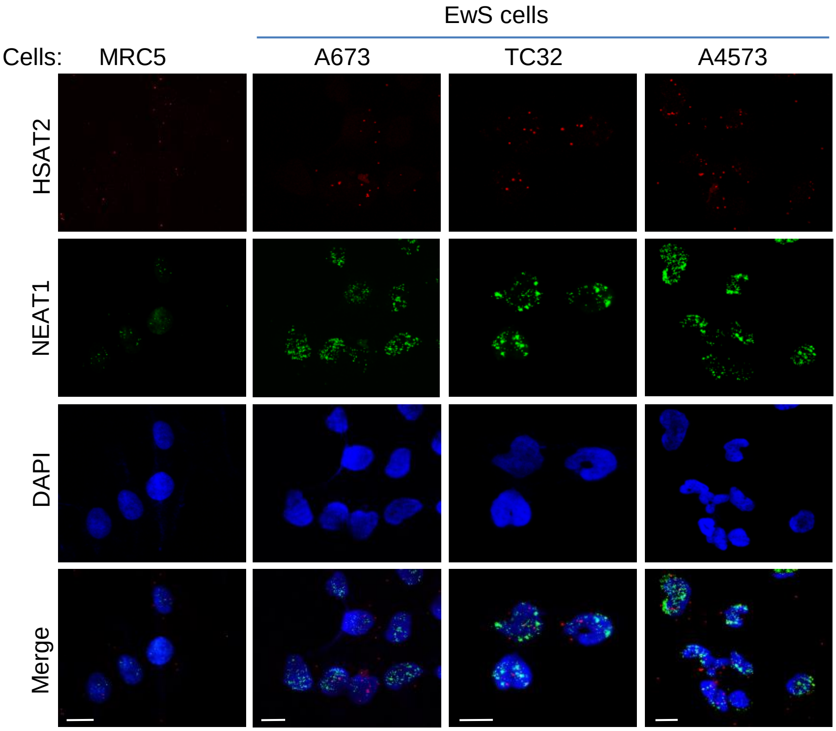

Supplemental Figure 10. *HSAT2* RNAs are localized to the nucleus and nuclear envelop in EwS cells. ViewRNA-FISH using *HSAT2* and *NEAT1* probes, counterstained with DAPI. Scale bars, 10 μm. Note that *HSAT2* is predominantly localized to the nuclear speckles distinct from the *NEAT1*-positive paraspeckles.

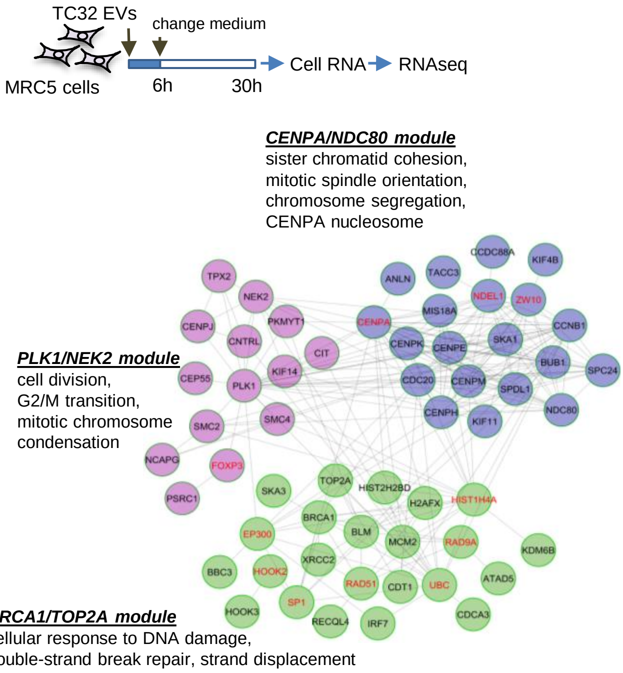

Supplemental Figure 11. EwS EVs activate innate immune and DNA damage pathways in the recipient cells. Outline of the experiment and Reactome functional interaction network analysis of mRNAs upregulated in TC32 EV-treated vs mock control MRC5 cells. The network consists of 46 (of total 54) mRNAs from the “Chromatin-remodeling”, “Cellular response to DNA damage” and “Cell division” GO categories, and 11 externally added linkers (red). Data are linked to Figure 7E and Supplemental Tables 3 and 4.

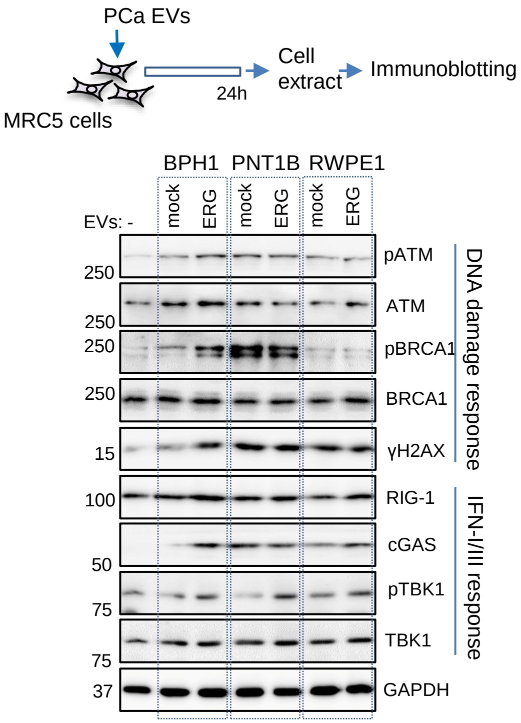

Supplemental Figure 12. *HSAT2*-enriched EVs from benign ERG expressing prostate cells activate pTBK1 signaling in the recipient MRC5 fibroblasts. Outline of the experiment and **i**mmunoblotting pathway analysis of MRC5 cells treated with PBS (-) or EVs from the indicated cell lines. Representative data from two independent experiments are shown. Note that pTBK1 signaling was activated by EVs from all 3 ERG expressing cell lines, while BPH1-ERG EVs also activated DNA damage response.
