# Supplementary material for: Oncogenic ETS fusions promote DNA damage and proinflammatory responses via pericentromeric RNAs in extracellular vesicles
Source: J Clin Invest. 2024 Mar 26;134(9):e169470. doi: 10.1172/JCI169470 (PMC11060741; doi:10.1172/JCI169470)
Supplement: Unedited blot and gel images [file jci-134-169470-s079.pdf]

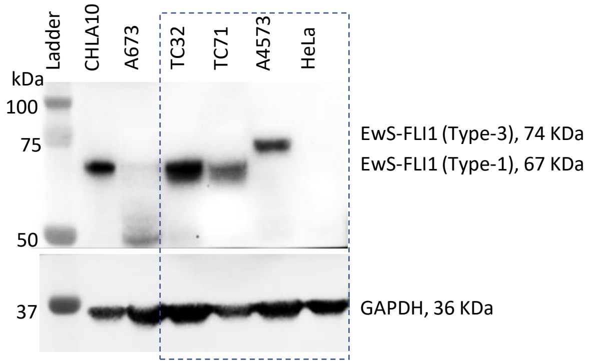

Full-size unedited gel for Figure 1A

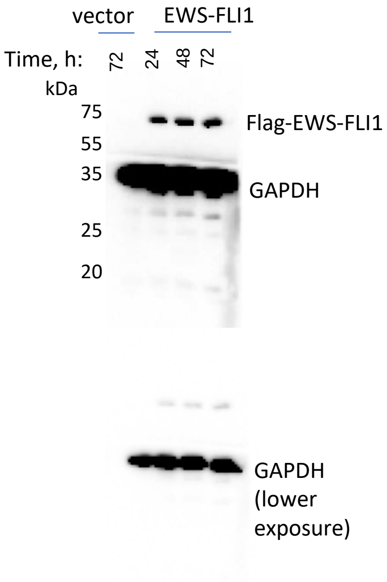

Full-size unedited gel for Figure 4D

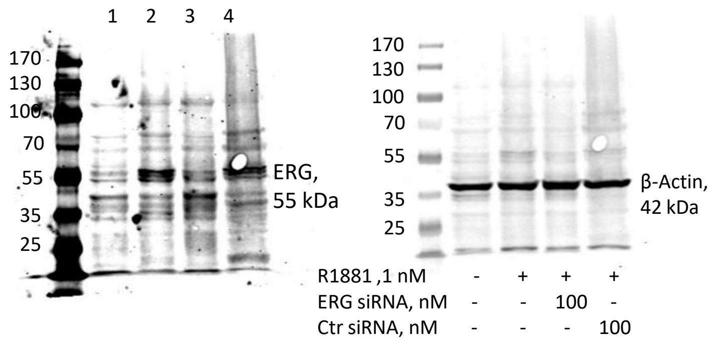

Full-size unedited gel for Figure 5C

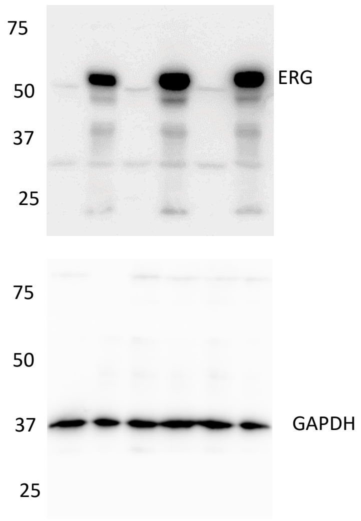

Full-size unedited gel for Figure 5G

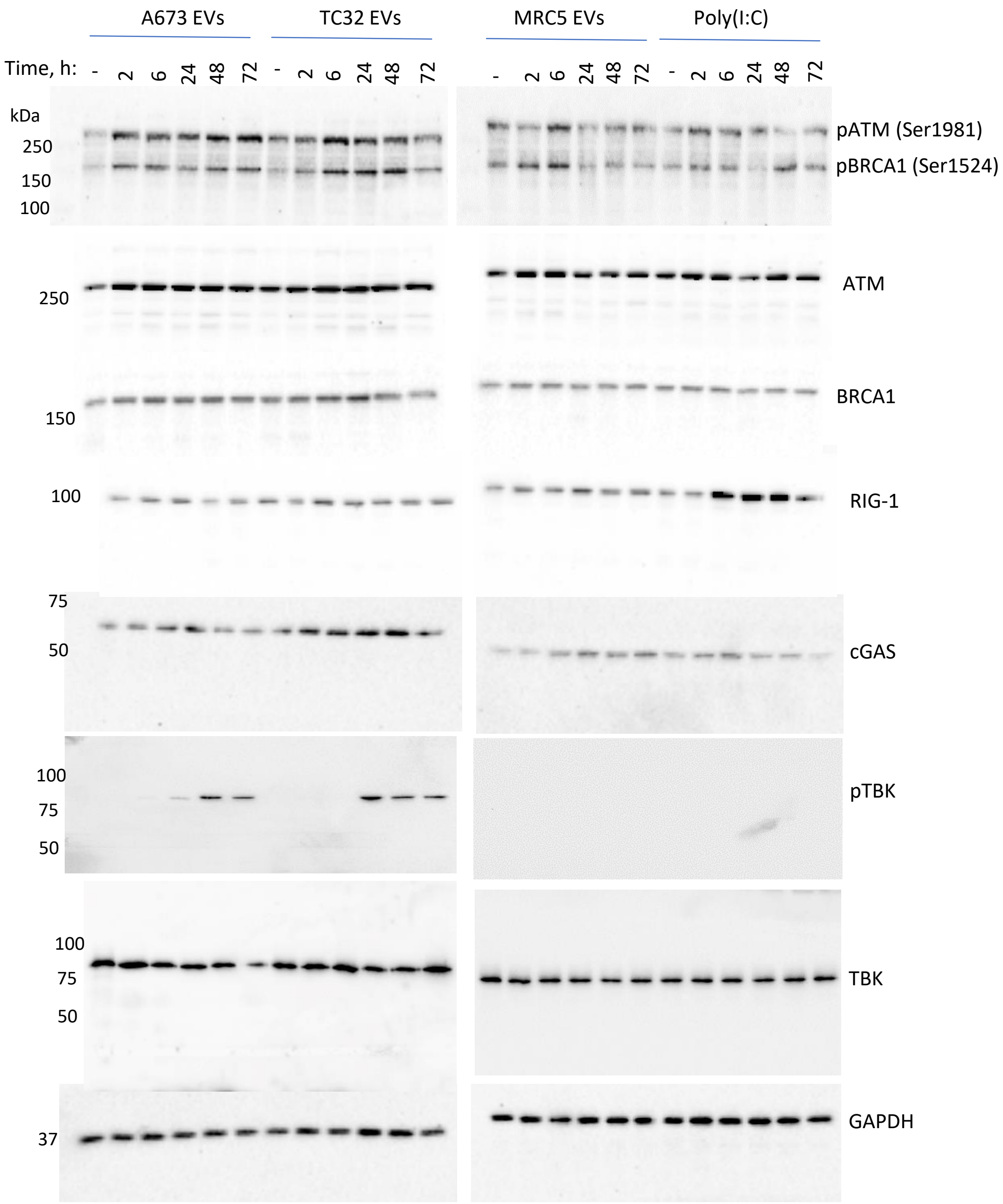

Full-size unedited gel for Figure 7F

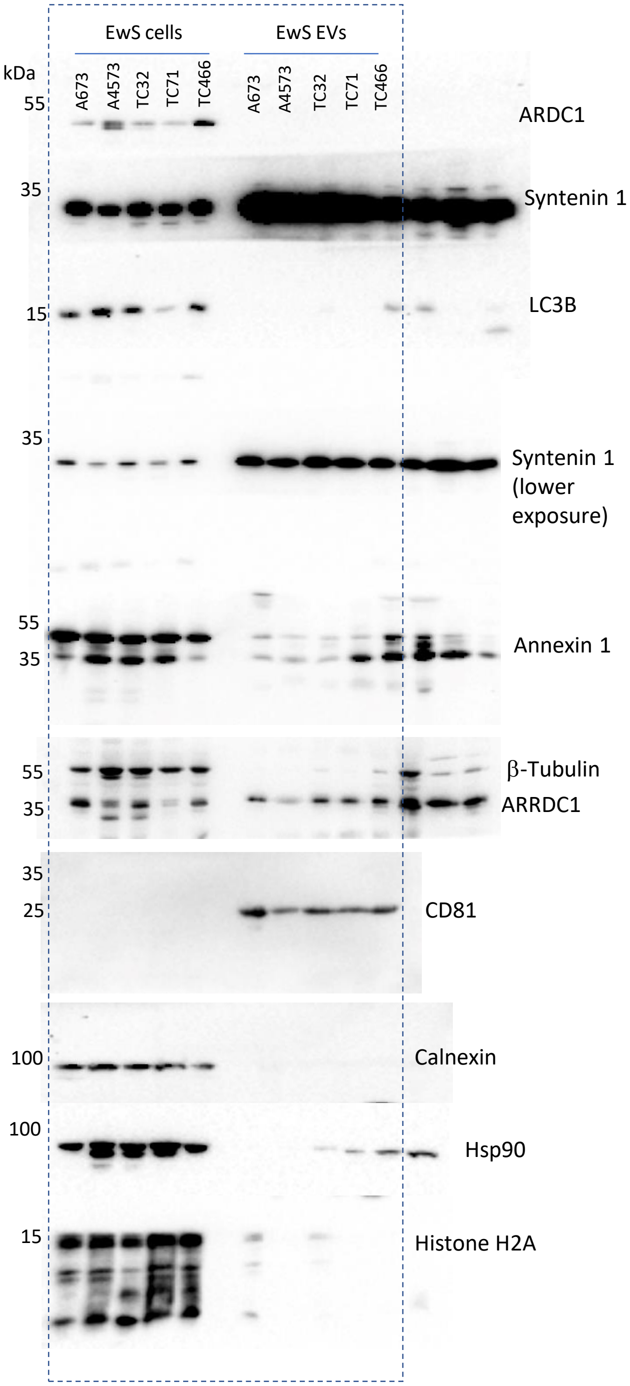

Full-size unedited gel for Supplemental Figure 1D

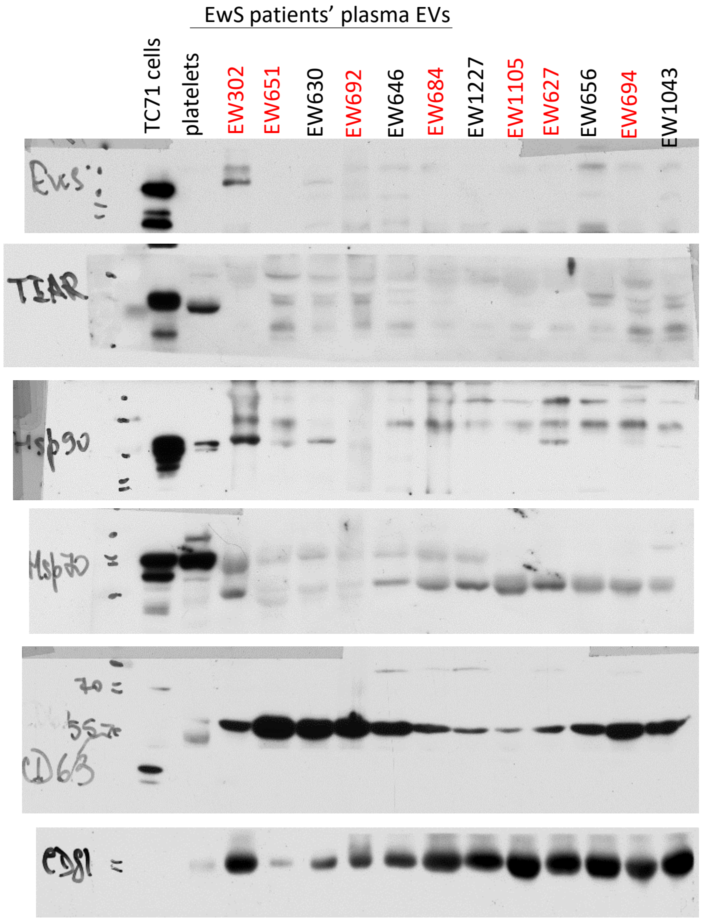

Full-size unedited gel for Supplemental Figure 2C

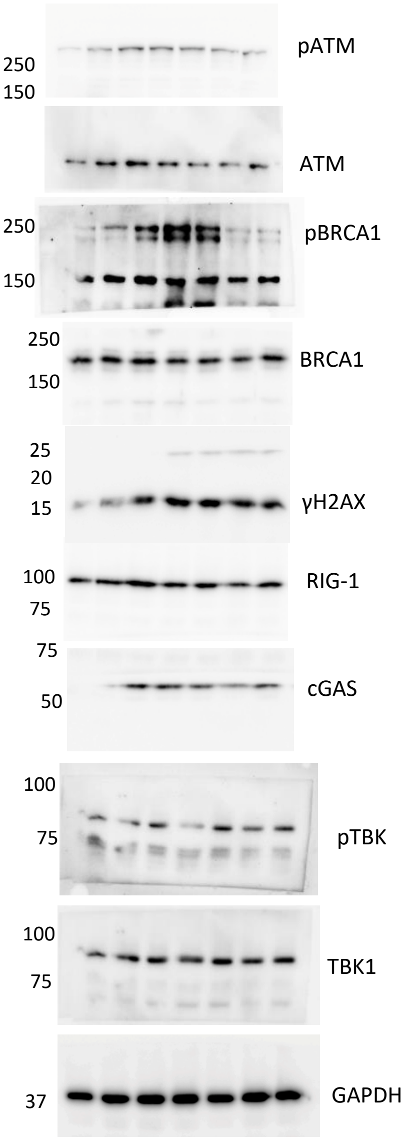

Full-size unedited gel for Supplemental Figure 12

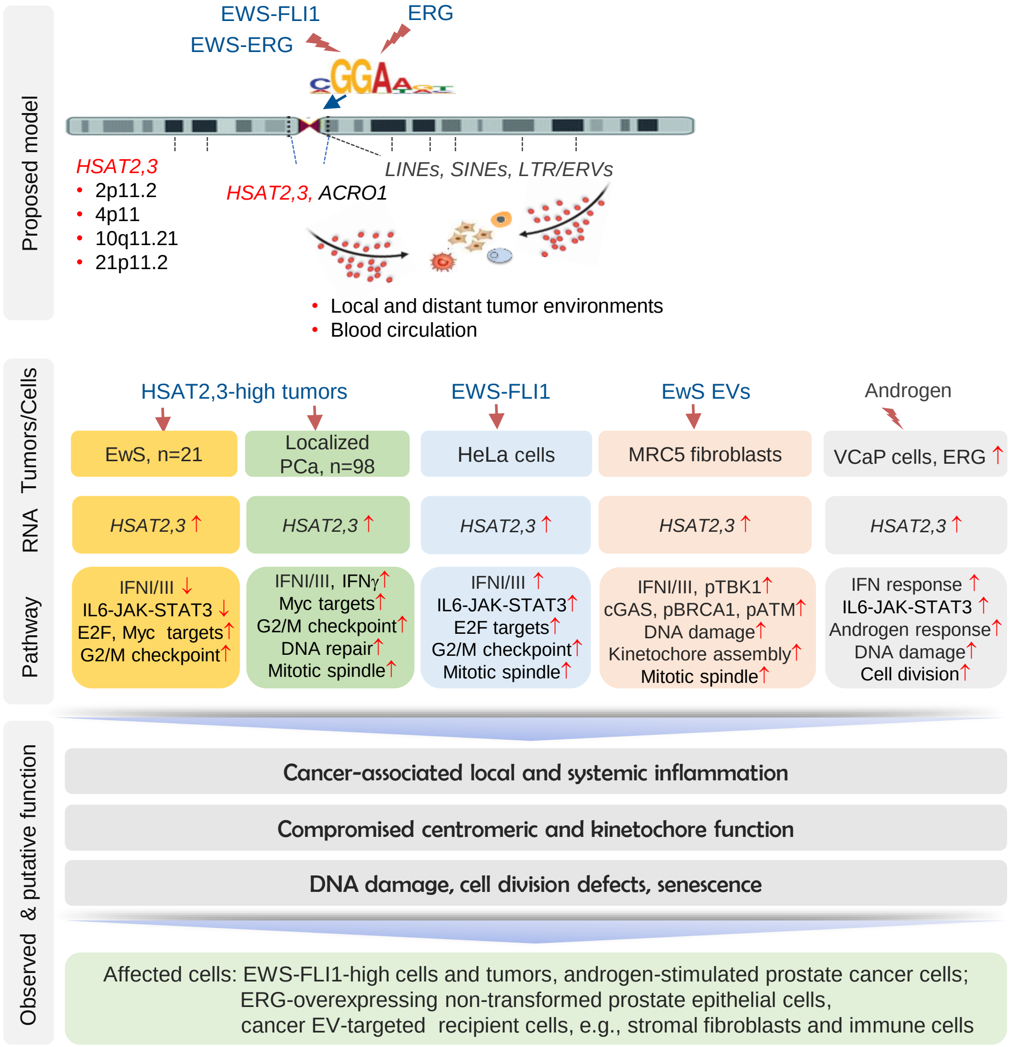
Proposed model
EWS-FLI1
EWS-ERG
ERG
HSAT2,3
2p11.2
4p11
10q11.21
21p11.2
HSAT2,3, ACRO1
LINEs, SINEs, LTR/ERVs
Local and distant tumor environments
Blood circulation
Tumors/Cells
RNA
Pathway
HSAT2,3-high tumors
EWS-FLI1
EwS EVs
Androgen
EwS, n=21
Localized PCa, n=98
HeLa cells
MRC5 fibroblasts
VCaP cells, ERG ↑
HSAT2,3 ↑
HSAT2,3 ↑
HSAT2,3 ↑
HSAT2,3 ↑
HSAT2,3 ↑
IFNI/III ↓
IL6-JAK-STAT3 ↓
E2F, Myc targets ↑
G2/M checkpoint ↑
IFNI/III, IFNγ ↑
Myc targets ↑
G2/M checkpoint ↑
DNA repair ↑
Mitotic spindle ↑
IFNI/III ↑
IL6-JAK-STAT3 ↑
E2F targets ↑
G2/M checkpoint ↑
Mitotic spindle ↑
IFNI/III, pTBK1 ↑
cGAS, pBRCA1, pATM ↑
DNA damage ↑
Kinetochore assembly ↑
Mitotic spindle ↑
IFN response ↑
IL6-JAK-STAT3 ↑
Androgen response ↑
DNA damage ↑
Cell division ↑
Observed & putative function
Cancer-associated local and systemic inflammation
Compromised centromeric and kinetochore function
DNA damage, cell division defects, senescence
Affected cells: EWS-FLI1-high cells and tumors, androgen-stimulated prostate cancer cells;
ERG-overexpressing non-transformed prostate epithelial cells,
cancer EV-targeted recipient cells, e.g., stromal fibroblasts and immune cells
